# Unraveling the associations of age and menopause with cardiovascular risk factors in a large population-based study

**DOI:** 10.1186/s12916-016-0762-8

**Published:** 2017-01-04

**Authors:** A. C. de Kat, V. Dam, N. C. Onland-Moret, M. J. C. Eijkemans, F. J. M. Broekmans, Y. T. van der Schouw

**Affiliations:** 1 Department of Reproductive Medicine and Gynecology, University Medical Center Utrecht, Heidelberglaan 100, Utrecht, 3508 GA The Netherlands; 2 Julius Center for Health Sciences and Primary Care, University Medical Center Utrecht, Heidelberglaan 100, Utrecht, 3508 GA The Netherlands

**Keywords:** Menopause, Cardiovascular risk, Female aging

## Abstract

**Background:**

Although the association between menopause and cardiovascular disease (CVD) risk has been studied extensively, the simultaneous role of chronological aging herein remains underexposed. This study aims to disentangle the relationships of menopausal status and chronological aging with CVD risk factors in the largest study population to date.

**Methods:**

In this cross-sectional study, CVD risk factors were compared between women with a different menopausal status within the same yearly age strata. The study population comprised female participants of the baseline visit of the population-based LifeLines Cohort Study. A total of 63,466 women, aged between 18 and 65 years, was included. Of them, 39,379 women were considered to be premenopausal, 8669 were perimenopausal, 14,514 were naturally postmenopausal, and 904 were surgically postmenopausal.

**Results:**

Compared to postmenopausal women aged 45 years, average total cholesterol (TC) and low-density lipoprotein cholesterol (LDL-c) were 0.5 and 0.4 mmol/L higher, respectively, in postmenopausal women aged 50. Systolic and diastolic blood pressure levels were 4 and 1 mmHg higher, respectively. At all ages between 46 and 55 years, and after adjustment for confounders, naturally postmenopausal women had 0.2 to 0.4 mmol/L higher TC and 0.1 to 0.3 mmol/L higher LDL-c levels compared to premenopausal women in the same age range. Systolic blood pressure levels were up to 4 mmHg lower in naturally post- compared to premenopausal women at all ages between 29 and 52 years. Body mass index levels were up to 3.2 kg/m^2^ higher in women with surgical menopause compared to all other women between the ages 32 and 52 years. All aforementioned results were statistically significant.

**Conclusions:**

Chronological age and menopausal status are both independently associated with CVD risk factors. Based on the comparatively smaller observed differences associated with menopausal status than with chronological aging, the significance of a more unfavorable lipid profile in a later reproductive stage may be less obvious than previously thought.

**Electronic supplementary material:**

The online version of this article (doi:10.1186/s12916-016-0762-8) contains supplementary material, which is available to authorized users.

## Background

Menopause is the final result of the continuous decline of ovarian reserve, marking the end of a woman’s reproductive lifespan. An earlier age of reaching menopause is considered to be associated with an increased risk of cardiovascular disease (CVD) [1, 2], but the mechanisms through which menopause is associated with CVD remain unclear. The menopausal transition and postmenopausal status have been associated with adverse CVD risk factor levels [3–10], but studies have recently contended that chronological aging or prior CVD risk play a more important role [11–13].

As postmenopausal women are, by definition, older than premenopausal women, it is challenging to separate the effects of biological aging from the various phases of the reproductive aging process [14]. This problem was previously circumvented by exclusively studying 53-year-old women born within the same week [7], longitudinally estimating the rate of change of CVD risk factors in the time surrounding the final menstrual period [6, 15], or comparing blood pressure levels between women in biannual age strata [16]. However, as the menopausal transition occurs over several years, its longitudinal effects can be ascribed to both aging and menopausal status in the same participant. The currently available studies were furthermore not able to assess the individual effects of chronological and reproductive aging over a large age interval.

In this study, we aimed to disentangle the associations of menopausal status and chronological aging with CVD risk factors over a wide age range. To this end, we compared levels of CVD risk factors with menopausal status, within and between yearly age strata, in the largest study population to date.

## Methods

### Cohort profile

For our study population, there were 80,853 potentially eligible women between 18 and 65 years old who participated in the baseline examination of the LifeLines Cohort Study. Lifelines is a multi-disciplinary prospective population-based cohort study examining, in a unique three-generation design, the health and health-related behaviors of 167,729 persons living in the north of The Netherlands. It employs a broad range of investigative procedures in assessing the biomedical, socio-demographic, behavioral, physical and psychological factors which contribute to the health and disease of the general population, with a special focus on multi-morbidity and complex genetics [17, 18]. The cohort participants were recruited through general practitioner registrations between 2006 and 2013. Cohort members are examined at baseline and will be prospectively followed up with visits in 5-year intervals and questionnaires every 1.5 years. The current study was based on information from the baseline examination, which includes a questionnaire, anthropometric measurements and blood withdrawal. All participants gave written informed consent [19] and ethical approval was granted by the medical ethics committee of University Medical Center Groningen [18]. LifeLines is a facility that is open for all researchers. Information on application and data access procedure is summarized on www.lifelines.net.

### Menopausal status assessment

Women with an intra-uterine contraceptive device (*n* = 2445, 3.0%), who previously underwent a hysterectomy (*n* = 4937, 6.2%), and/or who reported never having had a regular menstrual cycle (*n* = 4780, 5.9%) were excluded, leaving 73,662 women. Participants were then divided into groups based on menopausal status, which were defined as premenopausal, perimenopausal, naturally postmenopausal or surgically menopausal. Group allocation was based on baseline questionnaire information and followed the Stages of Reproductive Aging Workshop (STRAW) criteria [20]. Women with a currently regular menstrual cycle (*n* = 39,379, 53.4%) were classified as premenopausal. Women with an irregular menstrual cycle since several months (*n* = 7661) or years (*n* = 1260; total *n* = 8669, 11.8%) were considered to be perimenopausal. Women who answered that they were postmenopausal when asked about cycle regularity, and with a date of last menstruation being more than 1 year before the visit (*n* = 14,514, 19.7%), were considered to be naturally postmenopausal. Women who reported having had a bilateral oophorectomy (*n* = 904, 6.7%) were classified as surgically postmenopausal. The reproductive status of 5293 (7.2%) women could not be determined. This left 63,466 women in the study population.

### Cardiovascular risk factor assessment

At the baseline examination, height and weight were measured by trained staff, from which body mass index (BMI; in kg/m^2^) was calculated. Systolic and diastolic blood pressure (SBP and DBP) were measured 10 times during 10 minutes using a Dynamap PRO (GE Healthcare, Freiburg, Germany) [18], from which the average values were used. The baseline examination furthermore included fasting venous blood withdrawal. Directly after blood withdrawal, prespecified biomarkers in each fasting blood sample were routinely assessed at the in-house laboratory of the University Medical Center Groningen. Serum levels of total cholesterol (TC) and high-density lipoprotein cholesterol (HDL-c) were assessed with an enzymatic colorimetric method, low-density lipoprotein cholesterol (LDL-c) was assessed with a colorimetric method and triglyceride (TG) levels were measured with a colorimetric ultraviolet method, with a Roche Modular P chemistry analyzer (Roche, Basel, Switzerland). Fasting blood glucose was assessed with a hexokinase method [21].

### Other variables

The questionnaires additionally contained questions about hormonal contraception or postmenopausal hormone therapy (HT) use and smoking status. Participants were asked whether they had ever or were currently using oral contraceptives, a hormonal intrauterine device, contraceptive injection (henceforth altogether referred as hormonal contraception) or HT. Current use included any use in the prior month. Smoking status was assessed by asking participants whether they were current smokers or had smoked the previous months. Current and ever smokers were furthermore asked about the total duration and daily frequency of smoking. For this study, smoking status was defined as current smoker (yes or no), including women who had smoked up until the prior month.

Women who were pregnant at the time of examination (*n* = 109, 0.1%) were asked to fill out the questionnaire about the period preceding their pregnancy. They completed their baseline visit at least 6 months after their pregnancy and 3 months after ceasing to breastfeed, at which point the questionnaire was handed in and blood withdrawal occurred.

### Data analysis

For all variables of interest, the number of complete cases was 60,811 (96%) and missing information per variable did not exceed 1%. Missing values were imputed by conditional multiple imputation with 10 iterations, through predictive mean matching for continuous variables and proportional odds for categorical variables. All CVD risk factor variables, with the exception of TG, were normally distributed. As the distribution of TG levels was right-skewed, TG levels were log-transformed. Baseline characteristics were presented across menopausal status groups as mean ± SD or n (%), unless stated otherwise.

To gain a first insight in differences in CVD risk factor levels between the menopausal status categories independently of age, a linear regression analysis was performed within each 1-year age stratum for each outcome, with premenopausal women as the reference category. Women below the age of 34 were all included in a 34-years and younger group, due to the relative lack of postmenopausal women before this age. In a similar fashion, women above age of 56 were all included in a 56-years and older age stratum. The regression analyses were adjusted for smoking status, current hormonal contraception and BMI due to their potential association with both menopausal status and CVD risk factors. Because BMI was considered to be both a potential confounder and CVD risk factor, a model with BMI as an outcome was also fit, which adjusted for smoking and hormonal contraception use only. Models were furthermore adjusted for antihypertensive and lipid-lowering medication.

The objective of investigating an independent association of both calendar age and menopausal status with CVD risk factor levels was addressed by creating a linear regression model for each CVD risk factor as an outcome, with menopausal status and age as independent covariables. In order to adjust for smoking status, hormonal contraception use, antihypertensive or lipid-lowering medication and BMI (except in the case of BMI as a CVD risk factor outcome), these parameters were additionally added to the model. To test whether the association with age differed between the menopausal status groups, we included an interaction term of menopausal status with age in the model and tested its significance with an analysis of variance (ANOVA). Furthermore, in order to take into account a potential non-linear relationship of age with CVD risk factors, restricted cubic splines for age were added to the model [22, 23]. The model was then tested for non-linearity with an ANOVA analysis. Using the resulting best fitting model (excluding the interaction term or splines if the interaction term or test for non-linearity were non-significant), the adjusted values for each outcome were plotted against age for each menopausal status group.

All statistical analyses were performed with R (www.r-project.org), version 3.1.3. Multiple imputation was done using the ‘mice’ library, using a prediction matrix with all determinants, outcomes and confounders [24]. The regression models were fitted with the fit.mult.impute function from the ‘Hmisc’ library.

### Sensitivity analyses

We performed four sensitivity analyses. First, the analyses described above were repeated after including women with missing reproductive status information, by assigning them to menopausal groups based on their age, similar to the methods by Clavel-Chapelon et al. [25]. Secondly, the analyses were repeated after excluding women who reported current use of cholesterol- or blood pressure-lowering medication. Thirdly, the analyses were performed with only inclusion of women who reported an irregular cycle ‘since several months’ as the perimenopausal group. Finally, as the classification of the STRAW criteria for the whole study population was based on the answers to the question of cycle regularity and menopause, hormonal contraception and HT use were not taken into account for this determination. To assess the differences between the menopausal status groups independently from exogenous hormone use, women who had ever used HT or currently used hormonal contraception were excluded from analysis.

### Patient involvement

The development of the research question and study design occurred without the involvement of patients. The research question fits within the scope of healthy aging in the general population, an objective set by LifeLines.

## Results

In Table 1, the number of women in each age stratum and menopausal status group is listed. Characteristics for women in each reproductive category are presented in Table 2. Mean age increased over the pre-, peri-, and postmenopausal groups, and so did the mean levels of all CVD risk factors. Hormonal contraception usage decreased over the pre-, peri- and postmenopausal groups, with the lowest percentage of users in the surgically postmenopausal group. The vast majority of women who reported ever using HT (3% of the study population) were postmenopausal (77%), with the highest percentage (64%) in the surgical menopause group. In the premenopausal group, 203 (0.5%) women said to have ever used HT, but reported a currently regular menstrual cycle. In the naturally postmenopausal group, median age (interquartile range, IQR) at menopause was 51 (46–53) years.

**Table 1:** Number of study participants in each menopausal status group per annual age stratum

Age stratum	Premenopausal	Perimenopausal	Naturally postmenopausal	Surgically postmenopausal	Total
18	726	32	1	0	759
19	561	19	2	0	582
20	552	35	3	0	590
21	638	29	8	1	676
22	655	50	4	0	709
23	670	50	7	0	727
24	704	51	14	0	769
25	813	79	10	0	902
26	1138	119	26	1	1284
27	1064	120	25	0	1209
28	982	101	19	0	1102
29	971	119	19	1	1110
30	950	107	27	0	1084
31	995	116	28	2	1141
32	1028	119	42	3	1192
33	1070	110	32	1	1213
34	1118	99	39	2	1258
35	1151	113	54	1	1319
36	1235	138	70	5	1448
37	1369	134	83	8	1594
38	1464	145	63	6	1678
39	1606	153	108	15	1882
40	1731	187	95	7	2020
41	1796	245	119	11	2171
42	1860	248	121	16	2245
43	1783	352	135	22	2292
44	1781	380	137	19	2317
45	1740	490	157	28	2415
46	1577	535	208	40	2360
47	1489	659	290	39	2477
48	1337	743	392	43	2515
49	1148	788	508	61	2505
50	898	795	703	51	2447
51	467	565	691	37	1760
52	134	233	464	18	849
53	69	169	556	22	816
54	57	123	653	32	865
55	29	62	802	24	917
56	14	32	886	23	955
57	4	16	870	28	918
58	1	6	902	38	947
59	3	1	876	47	927
60	1	1	900	29	931
61	0	0	883	30	913
62	0	1	817	52	870
63	0	0	821	37	858
64	0	0	790	48	808
65	0	0	84	56	140
Total	39397	8669	14514	904	63466

**Table 2:** Characteristics per menopausal status group

	Premenopausal	Perimenopausal	Naturally postmenopausal	Surgically postmenopausal
	*n* = 39,397	*n* = 8669	*n* = 14,514	*n = 904*
Baseline				
Age, years	36.9 ± 8.1	45.0 ± 8.1	55.3 ± 7.4	52.7 ± 8.1
Age range, years	18–60	18–62	18–65	21-65
Current hormonal contraception use	18,526 (47.6)	1938 (22.7)	1787 (12.6)	825 (2.6)
Current smoker	8125 (21.0)	1969 (22.9)	2751 (19.1)	165 (18.4)
Antihypertensive medications	1559 (4.0)	608 (7.0)	2356 (20.3)	182 (20.3)
Lipid-lowering medications	388 (1.0)	178 (2.1)	1222 (8.4)	92 (10.2)
Ever HT use	203 (0.5)^a^	275 (3.2)	1315 (9.1)	253 (28.4)
Outcome				
BMI, kg/m^2^	25.2 ± 4.6	26.0 ± 4.9	26.2 ± 4.5	27.3 ± 5.0
SBP, mmHg	119 ± 13	121 ± 14	125 ± 16	126 ± 16
DBP, mmHg	71 ± 9	72 ± 9	73 ± 9	72 ± 9
TC, mmol/L	4.7 ± 0.8	5.0 ± 0.9	5.6 ± 1.0	5.5 ± 1.0
LDL-c, mmol/L	2.9 ± 0.8	3.1 ± 0.8	3.6 ± 0.9	3.5 ± 0.9
HDL-c, mmol/L	1.6 ± 0.4	1.6 ± 0.4	1.7 ± 0.4	1.6 ± 0.4
TG, mmol/L	1.0 ± 0.5	1.0 ± 0.6	1.1 ± 0.6	1.2 ± 0.7
Glucose, mmol/L	4.8 ± 0.6	4.9 ± 0.7	5.0 ± 0.8	5.1 ± 1.0

Values given in mean ± SD or n (%)

^a^All reported a currently regular cycle

*HT* hormone replacement therapy, *BMI* body mass index, *SBP* systolic blood pressure; *DBP* diastolic blood pressure, *TC* total cholesterol, *LDL-c* low-density lipoprotein cholesterol, *HDL-c* high-density lipoprotein cholesterol, *TG* triglycerides

**Fig. 1:**
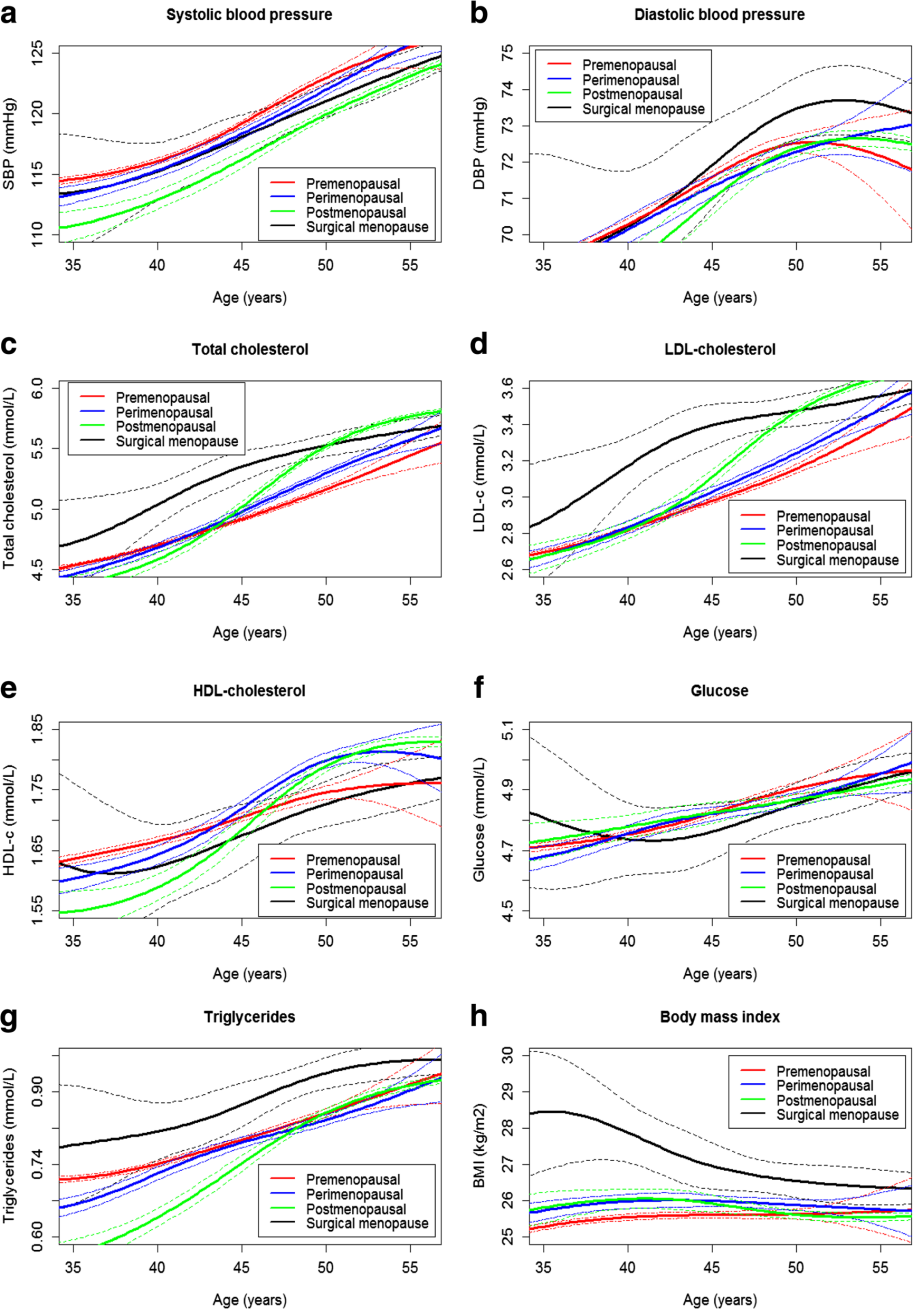
**a**–**h** Associations of adjusted cardiovascular risk factors with age per menopausal status group. Cardiovascular risk factor levels were adjusted for age, oral contraceptive use, smoking status and body mass index. The premenopausal status group comprised a total of 39,379 women, whereas the perimenopausal group comprised 8669 women, the naturally postmenopausal group 14,514 women, and the surgically postmenopausal group 904 women. **a** Systolic blood pressure, **b** Diastolic blood pressure, **c** Total cholesterol, **d** LDL-cholesterol, **e** HDL-cholesterol, **f** Glucose, **g** Triglycerides and **h** Body mass index

For all CVD risk factors studied, the association between age and risk factor level was significantly non-linear (*P* value for non-linearity < 0.001 in all cases), so all models included restricted cubic splines for age. In addition, for all CVD risk factors besides SBP and glucose there was a significant interaction between age and menopausal status (*P* values for the interaction term ranged between < 0.001 and 0.01), indicating that the magnitude of the differences in these risk factor levels between menopausal status groups varied with age. The models including cubic splines and the interaction term had a better fit than the models without, assessed by comparison of the Akaike’s Information Criterion. All model residuals were furthermore normally distributed. Since a single regression coefficient cannot be estimated due to the splines and interactions, the fully adjusted mean levels with 95% confidence interval (CI) bands of all CVD risk factors are displayed for each menopausal status group with age in Fig. 1a–h.

Between ages 29 and 52 mean SBP levels adjusted for hormonal contraception use, smoking and BMI were significantly lower in the naturally postmenopausal group compared to the three other menopausal status groups, as there was no overlap of CIs (Fig. 1a). Compared to the premenopausal group, fully adjusted SBP levels were between 2.6 and 4.0 mmHg lower in the naturally postmenopausal group. Similar results were found with the regression analyses within each age stratum (Additional file 1: Table S1 displays the regression coefficients with 95% CI for the linear regression analyses in each age stratum for SBP). With regard to chronological aging, compared to age 45, adjusted SBP levels at age 50 were between 3.0 to 3.8 mmHg higher on average (Table 3). No distinct pattern of differences between menopausal stages within the age bands was observed for DBP (Fig. 1b, Additional file 1: Table S2). Adjusted DBP levels in all menopausal status groups were between 0.9 and 1.6 mmHg higher at age 50 compared to age 45 (Table 3).

**Table 3:** Average absolute differences in adjusted risk factors between women aged 45 and 50 years

	Premenopausal	Perimenopausal	Naturally postmenopausal	Surgically menopausal
Difference in adjusted risk factor levels (95% CI) between women aged 45 and 50 years				
SBP, mmHg	3.8 (3.6 to 3.9)	3.6 (3.6 to 3.7)	3.7 (3.4 to 4.1)	3.0 (2.5 to 3.5)
DBP, mmHg	0.9 (0.8 to 1.0)	1.0 (1.0 to 1.0)	1.4 (1.2 to 1.6)	1.6 (1.2 to 1.9)
TC, mmol/L	0.2 (0.2 to 0.3)	0.3 (0.3 to 0.3)	0.5 (0.5 to 0.5)	0.2 (0.1 to 0.2)
LDL-c, mmol/L	0.2 (0.2 to 0.2)	0.2 (0.2 to 0.2)	0.4 (0.3 to 0.4)	0.1 (0.0 to 0.1)
HDL-c, mmol/L	0.0 (0.0 to 0.0)	0.1 (0.1 to 0.1)	0.1 (0.1 to 0.1)	0.1 (0.0 to 0.1)
Glucose, mmol/L	0.1 (0.1 to 0.1)	0.0 (0.0 to 0.0)	0.0 (0.0 to 0.1)	0.1 (0.1 to 0.1)
TG, mmol/L	0.1 (0.1 to 0.1)	0.1 (0.1 to 0.1)	0.1 (0.1 to 0.1)	0.1 (0.1 to 0.1)
BMI, kg/m^2^	0.0 (–0.0 to 0.1)	−0.1 (–0.1 to –0.1)	−0.3 (–0.4 to –0.2)	−0.4 (–0.6 to –0.2)

*SBP* systolic blood pressure, *DBP* diastolic blood pressure, *TC* total cholesterol, *LDL-c* low-density lipoprotein cholesterol, *HDL-c* high-density lipoprotein cholesterol, *TG* triglycerides, *BMI* body mass index

Fully adjusted mean TC and LDL-c levels were 0.1 mmol/L higher in the perimenopausal group compared to the premenopausal group, and 0.2–0.4 mmol/L higher in the naturally postmenopausal group compared to the premenopausal group across the range of 45–55 years, which reached statistical significance (Fig. 1c). Between 37 and 49 years, adjusted TC levels were 0.2–0.4 mmol/L higher in the surgically postmenopausal group compared to women in the premenopausal group, and significantly higher than all three other groups (Fig. 1c). Between 46 and 55 years, adjusted LDL-c levels in the peri- and naturally postmenopausal groups were 0.1 and 0.3 mmol/L, respectively. Surgically postmenopausal women had significantly higher adjusted LDL-c levels than all other women between the ages of 38 and 49. Linear regression analyses within the age strata echoed these results (Additional file 1: Table S3 and Table S4). With respect to chronological aging, the average adjusted difference in TC and LDL-c levels between 45 and 50 years ranged from 0.2 to 0.5 and 0.2 to 0.4 mmol/L, respectively (Table 3).

No clear differences were observed in mean adjusted HDL-c or glucose levels between the menopausal status groups at all ages (Fig. 1e, f, Additional file 1: Table S5 and Table S6). Compared to women aged 45 years, mean adjusted HDL-c and glucose levels were 0.0–0.1 mmol/L higher at age 50, dependent on menopausal status group (Table 3).

Fully adjusted mean TG levels were up to 12% higher in surgically postmenopausal women compared to premenopausal women between the ages 42 and 53. Between the ages 32 and 52, BMI levels were up to 3.2 kg/m^2^ higher in surgically postmenopausal compared to premenopausal women. In these age ranges, TG and BMI levels were significantly higher in surgically postmenopausal women compared to women in all other menopausal status groups (Fig. 1 g, h). In contrast, compared to premenopausal women, TG levels were 5–22% lower in postmenopausal women between the ages of 30 and 48. Similar results were found in the linear regression analyses in each age stratum, although the differences with the surgically postmenopausal group were not significant, possibly due to lack of power (Additional file 1: Table S5 and Table S6). At age 50, TG levels were 0.1 mmol/L higher in all menopausal status groups compared to age 45 (Table 3). Adjusted BMI levels were either the same or between 0.1 and 0.4 kg/m^2^ lower at age 50 compared to age 45, depending on the menopausal status group (Table 3).

### Sensitivity analyses

The sensitivity analyses are summarized for each outcome in Additional files 2, 3, 4, 5, 6, 7, 8 and 9: Figures S1–Figure S8. First, inclusion of the 5293 women with an age-based reproductive status did not alter the results. Second, the exclusion of women who used cholesterol- or blood pressure-lowering medication (*n* = 1880 and *n* = 4705, respectively) also did not alter the results, although the confidence interval of the surgical menopause group became wider. Third, excluding 1260 women in the perimenopausal group with an irregular cycle since several years additionally did not alter the nature of the results for the perimenopausal group. Fourth, exclusion of women using hormonal contraception (*n* = 23,076) and HT (*n* = 2056) caused an expected widening of the confidence intervals due to the reduced power. This did not affect the overall results, with the exception of a more marked difference in TC, LDL-c and TG levels between young pre- and postmenopausal women (Additional files 4, 5 and 7: Figures S3, Figure S4 and Figure S6).

## Discussion

This study presents a unique view of reproductive aging, independently of biological aging. We observed an association of CVD risk factors with menopausal status within several clusters of annual age strata, indicating that this relationship cannot be explained by the effects of chronological aging alone. The magnitude of differences in CVD risk factors between menopausal status groups did vary with age, highlighting the added role of chronological aging. Based on these results, it seems likely that both chronological aging and menopausal status contribute to the CVD risk profile of aging women.

Naturally postmenopausal women had lower adjusted SBP levels across a large age range than pre-, peri or surgically postmenopausal women. Prior reports found a later reproductive stage to be associated with increased blood pressure [9, 16, 26], while others reported a lack of any association after adjustment for age [6, 7, 13, 27, 28]. A longitudinal study in 193 women was the first to detect a decreased SBP level in post- compared to premenopausal women [29], hypothesizing that a diminishing ovarian reserve exhibits a protective effect on increasing SBP levels. By design, we cannot confirm this hypothesis, but our results do contest previous reports of an adverse blood pressure milieu in a peri- and postmenopausal state [9, 16, 26].

Where lipid levels are concerned, previous findings are less ambiguous and correspond well to our results. LDL-c and TC levels are widely thought to be influenced by the menopausal transition [6] or associated with menopausal status [4, 5, 7, 10, 30–33]. In fact, the approximate difference in LDL-c levels of 11 mg/dL (0.28 mmol/L) observed by Matthews et al. [6] between the year preceding and following the final menstrual period fits well within the range of our observations. The decrease of estradiol throughout the menopausal transition may not play a role in this regard, as TC and LDL-c levels did not correlate with total or free estradiol in 99 postmenopausal women [34]. On the other hand, post-menopausal hormone therapy was associated with a better lipid profile compared to placebo in a meta-analysis of 28 trials [35]. Another explanation is the reduced activity of LDL-c receptors or lipoprotein lipase in a postmenopausal state [36, 37].

In our population, differences in LDL-c and TC levels between menopausal status groups only became evident after the age of 45, after which LDL-c and TC levels more sharply increased in the peri- and postmenopausal groups. While a rapid increase in lipid levels was previously linked to the menopausal transition [4, 6], our results do suggest that chronological aging is equally involved. Indeed, the adjusted difference in TC and LDL-c values in the interval of 45–50 years was equal to the maximum observed differences between the menopausal status groups. It may be possible that, with increasing age, the availability of compensatory mechanisms to neutralize hyperlipidemia diminishes.

Surgically postmenopausal women, having undergone a bilateral oophorectomy, had consistently higher BMI and TG levels than the remaining women in the same age stratum, the latter even after adjusting for BMI. Others observed similar results [13, 38–42], with the odds of becoming obese specifically increasing after bilateral oophorectomy [41]. Interestingly, the adjusted BMI of pre-, peri- and naturally postmenopausal women hardly differed throughout the study population, which is in line with previous findings [38], but at odds with the observation that the menopausal transition influences fat distribution [15, 32].

For the past two decades, the relationship of menopause with CVD risk factors has been studied extensively through a myriad of ways. As most previous research was based on smaller study populations, often with significantly differing ages between pre- and postmenopausal groups, we hope to provide a substantial contribution to this age-old question with our study. Its strengths are the use of a large study population, with the ability to compare menopausal status groups and CVD risk factors within yearly age strata, over a wide age range. The protocolled assessment of study parameters and relative lack of missing information limit the chance of bias. Unfortunately, this was not quite the case for the classification of menopausal status. It is likely that some postmenopausal women using hormonal contraception or HT were classified as premenopausal due to the report of a regular cycle, and that some premenopausal women with an irregular cycle were wrongly classified as peri- or postmenopausal [43]. The exclusion of women using exogenous hormones did not have an obvious impact on the overall results, with the exception that the lipid profile of young postmenopausal women appeared notably more unfavorable than the other groups in this analysis. It is possible that this difference is due to the putative benefits of hormone supplementation in young women in particular [44], or incorrect classification of premenopausal women using hormonal contraception as postmenopausal. In order to be considered postmenopausal, women had to report in the questionnaire that they had entered menopause in addition to reporting an amenorrhea of at least a year, which makes large-scale misclassification in this category less likely. Moreover, the finding of very young women with non-iatrogenic menopause corresponds to our observations in clinical practice and other Dutch cohort studies and could therefore well be a realistic representation. Finally, due to the small number of women with surgical menopause, there is insufficient power to separately compare this group in all yearly strata. However, as this group of women represents a different clinical entity than natural menopause, we chose to maintain this classification.

As this was a cross-sectional study, our observations are limited to associations without drawing conclusions on causality. A previous study was able to longitudinally follow CVD risk factors [6], providing important information on the changes surrounding the menopausal transition. It is by definition impossible to distinguish these changes from general aging throughout the menopausal transition in the same participant, however, which is why our current study provides an important contribution from a different perspective. Although we are able to confirm previous reports of differences in lipid parameters based on menopausal status, the clinical implications of the observed differences may be limited. A reduction of LDL-c of 1.0 mmol/L was associated with a 22% decreased rate of major vascular events in an extensive meta-analysis of individual patient data [45], but this is difference is 2.5 to 10 times larger than menopause-related differences in this study or the study by Matthews et al. [6], and in fact more approximate to the differences found with 20 years of chronological age. It may be that the increased risk of cardiovascular events observed in post-menopausal women, the causality of which is a matter of debate in itself [11, 12, 46], is mediated through other pathways such as oxidative stress and inflammation [47]. A previous proposal of lipid screening of women entering the menopausal transition [6] may therefore not prove beneficial. That being said, vigilance of changing lipid parameters in high-risk women as they pass both biological and reproductive aging thresholds may be worthwhile.

## Conclusions

In conclusion, we observed independent associations of both age and menopausal status with selected CVD risk factors, mainly at the level of lipid metabolism, in a large population-based cohort. The clinical ramifications of a more unfavorable CVD risk factor profile with the transition to menopause may be limited, however.

## Additional files

Additional file 1: Table S1. Differences (95% CI) in adjusted systolic blood pressure levels (mmHg) per age stratum, in reference to premenopausal participants. **Table S2.** Differences (95% CI) in adjusted diastolic blood pressure levels (mmHg) per age stratum, in reference to premenopausal participants. **Table S3.** Differences (95% CI) in adjusted total cholesterol levels (mmol/L) per age stratum, in reference to premenopausal participants. **Table S4.** Differences (95% CI) in adjusted LDL-cholesterol levels (mmol/L) per age stratum, in reference to premenopausal participants. **Table S5.** Differences (95% CI) in adjusted HDL-cholesterol levels (mmol/L) per age stratum, in reference to premenopausal participants. **Table S6.** Differences (95% CI) in adjusted glucose levels (mmol/L) per age stratum, in reference to premenopausal participants. **Table S7.** Proportional differences (95% CI) in adjusted _log_triglyceride levels (mmol/L) per age stratum, in reference to premenopausal participants. **Table S8.** Differences (95% CI) in adjusted BMI levels (kg/m^2^) per age stratum, in reference to premenopausal participants. (DOCX 40 kb)
Additional file 2:
**Figure S1.** Sensitivity analyses of associations of systolic blood pressure with age per menopausal status group. From left to right: analyses with inclusion of women with missing reproductive status; analyses with exclusion of women using antihypertensive or lipid-lowering drugs; analyses with exclusion of women with an irregular cycle since several months or years; analyses with exclusion of women using exogenous hormones. (JPG 337 kb)
Additional file 3:
**Figure S2.** Sensitivity analyses of associations of diastolic blood pressure with age per menopausal status group. From left to right: analyses with inclusion of women with missing reproductive status; analyses with exclusion of women using antihypertensive or lipid-lowering drugs; analyses with exclusion of women with an irregular cycle since several months or years; analyses with exclusion of women using exogenoushormones. (JPG 640 kb)
Additional file 4:
**Figure S3.** Sensitivity analyses of associations of total cholesterol with age per menopausal status group. From left to right: analyses with inclusion of women with missing reproductive status; analyses with exclusion of women using antihypertensive or lipid-lowering drugs; analyses with exclusion of women with an irregular cycle since several months or years; analyses with exclusion of women using exogenous hormones. (JPG 350 kb)
Additional file 5:
**Figure S4.** Sensitivity analyses of associations of low-density lipoprotein cholesterol with age per menopausal status group. From left to right: analyses with inclusion of women with missing reproductive status; analyses with exclusion of women using antihypertensive or lipid-lowering drugs; analyses with exclusion of women with an irregular cycle since several months or years; analyses with exclusion of women using exogenous hormones. (JPG 352 kb)
Additional file 6:
**Figure S5.** Sensitivity analyses of associations of high-density lipoprotein cholesterol with age per menopausal status group. From left to right: analyses with inclusion of women with missing reproductive status; analyses with exclusion of women using antihypertensive or lipid-lowering drugs; analyses with exclusion of women with an irregular cycle since several months or years; analyses with exclusion of women using exogenous hormones. (JPG 640 kb)
Additional file 7:
**Figure S6.** Sensitivity analyses of associations of glucose with age per menopausal status group. From left to right: analyses with inclusion of women with missing reproductive status; analyses with exclusion of women using antihypertensive or lipid-lowering drugs; analyses with exclusion of women with an irregular cycle since several months or years; analyses with exclusion of women using exogenous hormones. (JPG 345 kb)
Additional file 8:
**Figure S7.** Sensitivity analyses of associations of triglycerides with age per menopausal status group. From left to right: analyses with inclusion of women with missing reproductive status; analyses with exclusion of women using antihypertensive or lipid-lowering drugs; analyses with exclusion of women with an irregular cycle since several months or years; analyses with exclusion of women using exogenous hormones. (JPG 324 kb)
Additional file 9:
**Figure S8.** Sensitivity analyses of associations of body mass index with age per menopausal status group. From left to right: analyses with inclusion of women with missing reproductive status; analyses with exclusion of women using antihypertensive or lipid-lowering drugs; analyses with exclusion of women with an irregular cycle since several months or years; analyses with exclusion of women using exogenous hormones. (JPG 315 kb)

## Abbreviations

BMI: Body mass index
CI: Confidence interval
CVD: Cardiovascular disease
DBP: Diastolic blood pressure
HDL-c: High-density lipoprotein cholesterol
LDL-c: Low-density lipoprotein cholesterol
SBP: Systolic blood pressure
TC: Total cholesterol
TG: Triglyceride
