# Erratum to: Unraveling the associations of age and menopause with cardiovascular risk factors in a large population-based study

**DOI:** 10.1186/s12916-017-0841-5

**Published:** 2017-03-29

**Authors:** A. C. de Kat, V. Dam, N. C. Onland-Moret, M. J. C. Eijkemans, F. J. M. Broekmans, Y. T. van der Schouw

**Affiliations:** 1 Department of Reproductive Medicine and Gynecology, University Medical Center Utrecht, Heidelberglaan 100, Utrecht, 3508 GA The Netherlands; 2 Julius Center for Health Sciences and Primary Care, University Medical Center Utrecht, Heidelberglaan 100, Utrecht, 3508 GA The Netherlands

## Erratum

After publication of the original article [1], it came to the authors’ attention that a source of funding for V. Dam had been inadvertently omitted. The following sentence should have been included in the Funding section: “V. Dam is supported by grant 2013T083 from the Dutch Heart Foundation.”
