# Supplementary material for: Unraveling the associations of age and menopause with cardiovascular risk factors in a large population-based study
Source: BMC Med. 2017 Jan 4;15:2. doi: 10.1186/s12916-016-0762-8 (PMC5210309; doi:10.1186/s12916-016-0762-8)
Supplement: Additional file 1: Table S1. — Differences (95% CI) in adjusted systolic blood pressure levels (mmHg) per age stratum, in reference to premenopausal participants. Table S2. Differences (95% CI) in adjusted diastolic blood pressure levels (mmHg) per age stratum, in reference to premenopausal participants. Table S3. Differences (95% CI) in adjusted total cholesterol levels (mmol/L) per age stratum, in reference to premenopausal participants. Table S4. Differences (95% CI) in adjusted LDL-cholesterol levels (mmol/L) per age stratum, in reference to premenopausal participants. Table S5. Differences (95% CI) in adjusted HDL-cholesterol levels (mmol/L) per age stratum, in reference to premenopausal participants. Table S6. Differences (95% CI) in adjusted glucose levels (mmol/L) per age stratum, in reference to premenopausal participants. Table S7. Proportional differences (95% CI) in adjusted logtriglyceride levels (mmol/L) per age stratum, in reference to premenopausal participants. Table S8. Differences (95% CI) in adjusted BMI levels (kg/m2) per age stratum, in reference to premenopausal participants. (DOCX 40 kb) [file 12916_2016_762_MOESM1_ESM.docx]

Additional file 1

Table S1. Differences (95% CI) in adjusted **Systolic blood pressure** levels (mm Hg) per age stratum, in reference to premenopausal participants.

| **Age (years)** | **Premenopausal (reference)** | **Perimenopausal** | **Naturally postmenopausal** | **Surgically menopausal** |
| --- | --- | --- | --- | --- |
| ≤34 | 0.0 (Ref) | -1.5 (-2.0 to -0.9) | -3.6 (-4.7 to -2.4) | -0.16 (-6.4 to 5.9) |
| 35 | 0.0 (Ref) | -1.5 (-3.5 to 0.5) | -3.0 (-5.9 to -0.1) | 8.5 (-12.0 to 28.9) |
| 36 | 0.0 (Ref) | -2.3 (-4.2 to -0.5) | -2.7 (-5.3 to -0.1) | 2.3 (-6.8 to 11.5) |
| 37 | 0.0 (Ref) | -2.3 (-4.2 to -0.4) | -4.5 (-7.0 to -2.1) | 2.3 (-5.1 to 10.0) |
| 38 | 0.0 (Ref) | 0.6 (-1.3 to 2.6) | -5.0 (-7.9 to -2.0) | -2.2 (-11.4 to 7.0) |
| 39 | 0.0 (Ref) | -0.4 (-2.4 to 1.5) | -2.5 (-4.9 to -0.1) | -3.6 (-9.8 to 2.6) |
| 40 | 0.0 (Ref) | -0.7 (-2.5 to 1.1) | -2.6 (-5.2 to -0.1) | -6.2 (-15.2 to 2.7) |
| 41 | 0.0 (Ref) | -1.1 (-2.7 to 0.5) | -4.6 (-6.9 to -2.3) | -5.1 (-12.2 to 2.0) |
| 42 | 0.0 (Ref) | -1.2 (-2.8 to 0.5) | -2.7 (-5.1 to 0.4) | 0.5 (-5.7 to 6.7) |
| 43 | 0.0 (Ref) | -0.1 (-1.6 to 1.3) | -3.8 (-6.1 to -1.6) | 1.7 (-3.7 to 7.0) |
| 44 | 0.0 (Ref) | -1.0 (-2.5 to 0.5) | -3.1 (-5.5 to -0.8) | 1.2 (-4.9 to 7.2) |
| 45 | 0.0 (Ref) | 0.1 (-1.3 to 1.5) | -3.0 (-5.3 to -0.7) | -1.7 (-6.9 to 3.5) |
| 46 | 0.0 (Ref) | -1.0 (-2.4 to 0.3) | -3.7 (-5.7 to -1.7) | -3.2 (-7.5 to 1.1) |
| 47 | 0.0 (Ref) | -1.3 (-2.6 to -0.1) | -2.7 (-4.4 to -0.9) | -0.0 (-4.4 to 4.4) |
| 48 | 0.0 (Ref) | -2.2 (-3.6 to -0.9) | -2.5 (-4.1 to -0.9) | -0.9 (-5.2 to 3.5) |
| 49 | 0.0 (Ref) | -1.0 (-2.4 to 0.3) | -4.2 (-5.8 to -2.7) | -3.6 (-7.4 to 0.1) |
| 50 | 0.0 (Ref) | 0.2 (-1.2 to 1.7) | -1.6 (-3.1 to -0.1) | -1.6 (-5.8 to 2.5) |
| 51 | 0.0 (Ref) | -0.5 (-2.4 to 1.4) | -2.6 (-4.5 to -0.8) | -1.0 (-6.1 to 4.1) |
| 52 | 0.0 (Ref) | 0.6 (-2.7 to 3.9) | -2.2 (-5.2 to 0.8) | 2.6 (-4.9 to 10.2) |
| 53 | 0.0 (Ref) | -1.8 (-6.1 to 2.6) | -4.5 (-8.5 to -0.4) | -7.1 (-14.3 to 0.1) |
| 54 | 0.0 (Ref) | 1.3 (-3.5 to 6.2) | -1.7 (-6.0 to 2.7) | 1.7 (-4.8 to 8.1) |
| 55 | 0.0 (Ref) | -4.2 (-11.2 to 2.9) | -6.3 (-12.4 to -0.2) | -6.1 (-14.8 to 2.5) |
| ≥56 | 0.0 (Ref) | -1.5 (-9.7 to 6.8) | -1.9 (-9.1 to 5.2) | -0.5 (-7.8 to 6.8) |

Values reflect coefficients of linear regression analyses in each age stratum, adjusted for OC use, smoking status and BMI. Premenopausal women are the reference category. Red cells indicate p-value <0.0001, green cells indicate p-value <0.001, blue cells indicate p-value <0.05.

Table S2. Differences (95% CI) in adjusted **Diastolic blood pressure** levels (mm Hg) per age stratum, in reference to premenopausal participants.

| **Age (years)** | **Premenopausal (reference)** | **Perimenopausal** | **Naturally postmenopausal** | **Surgically menopausal** |
| --- | --- | --- | --- | --- |
| ≤34 | 0.0 (Ref) | -0.4 (-0.8 to -0.0) | -1.2 (-2.0 to -0.3) | 2.1 (-2.1 to 6.3) |
| 35 | 0.0 (Ref) | -0.8 (-2.3 to 0.6) | -1.4 (-3.5 to 0.7) | 2.9 (-11.7 to 17.6) |
| 36 | 0.0 (Ref) | -0.0 (-1.4 to 1.3) | -1.0 (-2.9 to -0.8) | 0.2 (-6.4 to 6.8) |
| 37 | 0.0 (Ref) | -0.9 (-2.3 to 0.5) | -2.3 (-4.1 to -0.5) | 0.2 (-5.1 to 5.6) |
| 38 | 0.0 (Ref) | 0.9 (-0.4 to 2.4) | -2.5 (-4.6 to -0.3) | 0.2 (-6.3 to 6.8) |
| 39 | 0.0 (Ref) | 0.7 (-0.6 to 2.1) | -1.5 (-3.1 to 0.2) | -2.0 (-6.3 to 2.3) |
| 40 | 0.0 (Ref) | -0.5 (-1.7 to 0.7) | -1.5 (-3.2 to 0.2) | -3.1 (-9.1 to 2.8) |
| 41 | 0.0 (Ref) | 0.1 (-1.0 to 1.3) | -2.4 (-4.1 to -0.8) | -4.0 (-9.0 to 0.5) |
| 42 | 0.0 (Ref) | -0.8 (-1.9 to 0.3) | -0.9 (-2.5 to 0.7) | 3.6 (-0.6 to 7.7) |
| 43 | 0.0 (Ref) | 0.0 (-1.0 to 1.0) | -0.4 (-1.9 to 1.1) | 3.0 (-0.6 to 6.6) |
| 44 | 0.0 (Ref) | -0.4 (-1.3 to 1.6) | -1.2 (-2.8 to 0.3) | 2.9 (-1.0 to 6.8) |
| 45 | 0.0 (Ref) | 0.2 (-0.7 to 1.1) | -0.7 (-2.2 to 0.7) | -0.3 (-3.6 to 3.0) |
| 46 | 0.0 (Ref) | -0.7 (-1.6 to 0.1) | -0.5 (-1.8 to 0.7) | -1.5 (-4.2 to 1.3) |
| 47 | 0.0 (Ref) | -0.8 (-1.6 to 0.0) | -0.7 (-1.8 to 1.4) | 0.6 (-2.2 to 3.5) |
| 48 | 0.0 (Ref) | -0.4 (-1.2 to 0.5) | 0.4 (-0.7 to 1.4) | -0.6 (-3.3 to 2.2) |
| 49 | 0.0 (Ref) | -0.8 (-1.6 to 0.1) | -0.9 (-1.9 to 0.0) | 0.3 (-2.1 to 2.7) |
| 50 | 0.0 (Ref) | 0.6 (-0.3 to 1.5) | 0.6 (-0.4 to 1.5) | 0.3 (-2.3 to 2.9) |
| 51 | 0.0 (Ref) | -0.8 (-1.9 to 0.4) | 0.0 (-1.1 to 1.2) | 2.0 (-1.0 to 5.1) |
| 52 | 0.0 (Ref) | 2.2 (0.2 to 4.2) | 1.5 (-0.3 to 3.3) | 3.2 (-1.3 to 7.7) |
| 53 | 0.0 (Ref) | -0.3 (-3.0 to 2.4) | -0.5 (-3.0 to 1.9) | -1.5 (-5.6 to 2.9) |
| 54 | 0.0 (Ref) | 1.4 (-1.6 to 4.4) | 0.5 (-2.2 to 3.2) | 3.0 (-1.0 to 7.0) |
| 55 | 0.0 (Ref) | -4.2 (-8.4 to 0.1) | -3.9 (-7.5 to -0.2) | -3.9 (-9.0 to 1.3) |
| ≥56 | 0.0 (Ref) | -0.3 (-4.8 to 4.2) | -0.2 (-4.2 to 3.7) | 0.6 (-3.4 to 4.6) |

Values reflect coefficients of linear regression analyses in each age stratum, adjusted for OC use, smoking status and BMI. Premenopausal women are the reference category. Green cells indicate p-value <0.001.

Table S3. Differences (95% CI) in adjusted **Total cholesterol** levels (mmol/L) per age stratum, in reference to premenopausal participants.

| **Age (years)** | **Premenopausal (reference)** | **Perimenopausal** | **Naturally postmenopausal** | **Surgically menopausal** |
| --- | --- | --- | --- | --- |
| ≤34 | 0.0 (Ref) | -0.1 (-0.1 to -0.1) | -0.2 (-0.3 to -0.1) | 0.1 (-0.4 to 0.7) |
| 35 | 0.0 (Ref) | -0.3 (-0.4 to -0.1) | -0.2 (-0.4 to 0.0) | -0.0 (-1.5 to 1.5) |
| 36 | 0.0 (Ref) | -0.1 (-0.2 to 0.1) | -0.2 (-0.4 to 0.0) | 0.6 (-0.1 to 1.2) |
| 37 | 0.0 (Ref) | 0.0 (-0.1 to 0.2) | -0.3 (-0.5 to -0.1) | 0.5 (-0.1 to 1.2) |
| 38 | 0.0 (Ref) | -0.1 (-0.2 to 0.1) | -0.3 (-0.5 to 0.1) | -0.2 (-0.8 to 0.5) |
| 39 | 0.0 (Ref) | 0.0 (-0.1 to 0.2) | -0.2 (-0.3 to -0.0) | 0.3 (-0.3 to 0.8) |
| 40 | 0.0 (Ref) | 0.0 (-0.1 to 0.2) | -0.1 (-0.3 to 0.1) | -0.2 (-0.8 to 0.4) |
| 41 | 0.0 (Ref) | 0.0 (-0.1 to 0.1) | -0.0 (-0.2 to 0.1) | 0.5 (0.0 to 1.0) |
| 42 | 0.0 (Ref) | -0.1 (-0.2 to 0.0) | -0.1 (-0.2 to 0.1) | 0.7 (0.3 to 1.1) |
| 43 | 0.0 (Ref) | 0.0 (-0.1 to 0.1) | 0.1 (-0.1 to 0.2) | 0.5 (0.1 to 0.8) |
| 44 | 0.0 (Ref) | -0.0 (-0.1 to 0.1) | 0.0 (-0.1 to 0.2) | 0.5 (0.1 to 0.8) |
| 45 | 0.0 (Ref) | 0.1 (0.0 to 0.2) | 0.1 (-0.0 to 0.3) | 0.6 (0.3 to 1.0) |
| 46 | 0.0 (Ref) | 0.1 (0.0 to 0.2) | 0.1 (-0.0 to 0.2) | 0.1 (-0.2 to 0.3) |
| 47 | 0.0 (Ref) | 0.0 (-0.0 to 0.1) | 0.3 (0.2 to 0.4) | 0.6 (0.3 to 0.9) |
| 48 | 0.0 (Ref) | 0.1 (0.1 to 0.2) | 0.3 (0.2 to 0.4) | 0.1 (-0.1 to 0.4) |
| 49 | 0.0 (Ref) | -0.0 (-0.1 to 0.1) | 0.3 (0.2 to 0.4) | 0.3 (0.0 to 0.5) |
| 50 | 0.0 (Ref) | 0.1 (-0.0 to 0.2) | 0.3 (0.2 to 0.4) | 0.3 (0.1 to 0.6) |
| 51 | 0.0 (Ref) | 0.1 (-0.1 to 0.2) | 0.3 (0.2 to 0.4) | 0.1 (-0.3 to 0.4) |
| 52 | 0.0 (Ref) | 0.2 (0.0 to 0.4) | 0.4 (0.2 to 0.6) | 0.2 (-0.2 to 0.7) |
| 53 | 0.0 (Ref) | 0.2 (-0.1 to 0.5) | 0.4 (0.1 to 0.6) | 0.5 (0.0 to 0.9) |
| 54 | 0.0 (Ref) | 0.0 (-0.3 to 0.3) | 0.2 (-0.1 to 0.5) | 0.3 (-0.1 to 0.8) |
| 55 | 0.0 (Ref) | -0.2 (-0.6 to 0.2) | 0.0 (-0.4 to 0.4) | -0.0 (-0.6 to 0.5) |
| ≥56 | 0.0 (Ref) | 0.2 (-0.3 to 0.7) | 0.5 (0.1 to 1.0) | 0.5 (0.1 to 0.9) |

Values reflect coefficients of linear regression analyses in each age stratum, adjusted for OC use, smoking status and BMI. Premenopausal women are the reference category. Red cells indicate p-value <0.0001, green cells indicate p-value <0.001, blue cells indicate p-value <0.05.

Table S4. Differences (95% CI) in adjusted **LDL-cholesterol** levels (mmol/L) per age stratum, in reference to premenopausal participants.

| **Age (years)** | **Premenopausal (reference)** | **Perimenopausal** | **Naturally postmenopausal** | **Surgically menopausal** |
| --- | --- | --- | --- | --- |
| ≤34 | 0.0 (Ref) | -0.1 (-0.1 to -0.0) | -0.1 (-0.2 to -0.0) | 0.2 (-0.3 to 0.7) |
| 35 | 0.0 (Ref) | -0.2 (-0.3 to -0.1) | -0.1 (-0.3 to 0.1) | -0.1 (-1.5 to 1.3) |
| 36 | 0.0 (Ref) | -0.0 (-0.2 to 0.1) | -0.0 (-0.2 to 0.1) | 0.4 (-0.2 to 1.0) |
| 37 | 0.0 (Ref) | 0.1 (-0.1 to 0.2) | -0.1 (-0.3 to 0.0) | 0.4 (-0.2 to 1.0) |
| 38 | 0.0 (Ref) | -0.0 (-0.2 to 0.1) | -0.1 (-0.3 to 0.1) | -0.1 (-0.7 to 0.5) |
| 39 | 0.0 (Ref) | 0.0 (-0.1 to 0.2) | -0.0 (-0.2 to 0.1) | 0.3 (-0.2 to 0.8) |
| 40 | 0.0 (Ref) | 0.0 (-0.1 to 0.1) | -0.1 (-0.2 to 0.1) | -0.3 (-0.9 to 0.3) |
| 41 | 0.0 (Ref) | 0.1 (-0.0 to 0.2) | 0.1 (-0.0 to 0.3) | 0.6 (0.1 to 1.0) |
| 42 | 0.0 (Ref) | -0.0 (-0.1 to 0.1) | 0.0 (-0.1 to 0.2) | 0.5 (0.1 to 0.9) |
| 43 | 0.0 (Ref) | 0.0 (-0.1 to 0.1) | 0.1 (-0.0 to 0.3) | 0.5 (0.2 to 0.8) |
| 44 | 0.0 (Ref) | -0.0 (-0.1 to 0.1) | 0.0 (-0.1 to 0.2) | 0.4 (0.1 to 0.8) |
| 45 | 0.0 (Ref) | 0.1 (0.0 to 0.2) | 0.2 (0.0 to 0.3) | 0.5 (0.3 to 0.8) |
| 46 | 0.0 (Ref) | 0.1 (0.0 to 0.2) | 0.1 (0.0 to 0.2) | 0.1 (-0.1 to 0.4) |
| 47 | 0.0 (Ref) | -0.0 (-0.1 to 0.7) | 0.2 (0.1 to 0.4) | 0.6 (0.3 to 0.9) |
| 48 | 0.0 (Ref) | 0.1 (0.0 to 0.2) | 0.2 (0.1 to 0.3) | 0.1 (-0.2 to 0.3) |
| 49 | 0.0 (Ref) | -0.0 (-0.1 to 0.1) | 0.3 (0.2 to 0.3) | 0.2 (0.0 to 0.5) |
| 50 | 0.0 (Ref) | 0.1 (-0.0 to 0.1) | 0.3 (0.2 to 0.4) | 0.3 (0.1 to 0.6) |
| 51 | 0.0 (Ref) | -0.0 (-0.1 to 0.1) | 0.3 (0.1 to 0.4) | 0.2 (-0.1 to 0.5) |
| 52 | 0.0 (Ref) | 0.2 (0.0 to 0.4) | 0.3 (0.1 to 0.5) | 0.1 (-0.3 to 0.6) |
| 53 | 0.0 (Ref) | 0.2 (-0.0 to 0.5) | 0.4 (0.1 to 0.6) | 0.5 (0.1 to 0.9) |
| 54 | 0.0 (Ref) | -0.0 (-0.3 to 0.3) | 0.1 (-0.2 to 0.4) | 0.3 (-0.2 to 0.7) |
| 55 | 0.0 (Ref) | -0.1 (-0.6 to 0.3) | 0.0 (-0.3 to 0.4) | -0.0 (-0.5 to 0.5) |
| ≥56 | 0.0 (Ref) | 0.2 (-0.3 to 0.7) | 0.5 (0.1 to 0.9) | 0.5 (0.0 to 0.9) |

Values reflect coefficients of linear regression analyses in each age stratum, adjusted for OC use, smoking status and BMI. Premenopausal women are the reference category. Red cells indicate p-value <0.0001, green cells indicate p-value <0.001, blue cells indicate p-value <0.05.

Table S5. Differences (95% CI) in adjusted **HDL-cholesterol** levels (mmol/L) per age stratum, in reference to premenopausal participants.

| **Age (years)** | **Premenopausal (reference)** | **Perimenopausal** | **Naturally postmenopausal** | **Surgically menopausal** |
| --- | --- | --- | --- | --- |
| ≤34 | 0.0 (Ref) | -0.0 (-0.0 to -0.0) | -0.0 (-0.1 to 0.0) | -0.0 (-0.2 to 0.2) |
| 35 | 0.0 (Ref) | -0.1 (-0.1 to 0.0) | -0.1 (-0.2 to 0.0) | -0.0 (-0.7 to 0.6) |
| 36 | 0.0 (Ref) | -0.0 (-0.1 to 0.1) | -0.1 (-0.2 to 0.0) | 0.1 (-0.2 to 0.4) |
| 37 | 0.0 (Ref) | -0.0 (-0.1 to 0.0) | -0.1 (-0.2 to -0.0) | 0.1 (-0.2 to 0.3) |
| 38 | 0.0 (Ref) | 0.0 (-0.1 to 0.1) | -0.1 (-0.2 to -0.1) | -0.1 (-0.4 to 0.2) |
| 39 | 0.0 (Ref) | -0.0 (-0.1 to 0.1) | -0.1 (-0.2 to -0.1) | 0.0 (-0.2 to 0.2) |
| 40 | 0.0 (Ref) | 0.0 (-0.0 to 0.1) | -0.0 (-0.1 to 0.0) | -0.1 (-0.4 to 0.1) |
| 41 | 0.0 (Ref) | -0.0 (-0.1 to 0.0) | -0.1 (-0.2 to -0.0) | -0.1 (-0.3 to 0.1) |
| 42 | 0.0 (Ref) | -0.0 (-0.1 to 0.0) | -0.1 (-0.1 to 0.0) | 0.2 (0.0 to 0.3) |
| 43 | 0.0 (Ref) | -0.0 (-0.1 to 0.0) | -0.0 (-0.1 to 0.0) | -0.1 (-0.2 to 0.1) |
| 44 | 0.0 (Ref) | -0.0 (-0.0 to 0.0) | -0.0 (-0.1 to 0.1) | -0.0 (-0.2 to 0.1) |
| 45 | 0.0 (Ref) | 0.0 (-0.0 to 0.1) | -0.0 (-0.1 to 0.0) | 0.0 (-0.1 to 0.2) |
| 46 | 0.0 (Ref) | 0.0 (-0.0 to 0.0) | 0.0 (-0.0 to 0.1) | -0.1 (-0.2 to 0.0) |
| 47 | 0.0 (Ref) | 0.0 (0.0 to 0.1) | 0.0 (-0.0 to 0.1) | -0.0 (-0.2 to 0.1) |
| 48 | 0.0 (Ref) | 0.0 (0.0 to 0.1) | 0.0 (-0.0 to 0.1) | -0.0 (-0.1 to 0.1) |
| 49 | 0.0 (Ref) | 0.0 (-0.0 to 0.1) | 0.0 (-0.0 to 0.1) | -0.1 (-0.2 to 0.0) |
| 50 | 0.0 (Ref) | 0.0 (0.0 to 0.1) | 0.0 (-0.0 to 0.1) | -0.1 (-0.2 to 0.0) |
| 51 | 0.0 (Ref) | 0.1 (0.0 to 0.1) | 0.0 (-0.0 to 0.1) | -0.1 (-0.3 to 0.0) |
| 52 | 0.0 (Ref) | 0.0 (-0.1 to 0.1) | 0.0 (-0.0 to 0.1) | -0.0 (-0.2 to 0.2) |
| 53 | 0.0 (Ref) | 0.0 (-0.1 to 0.1) | 0.1 (-0.1 to 0.2) | 0.1 (-0.1 to 0.3) |
| 54 | 0.0 (Ref) | 0.1 (-0.1 to 0.2) | 0.1 (-0.0 to 0.2) | 0.1 (-0.1 to 0.3) |
| 55 | 0.0 (Ref) | -0.1 (-0.2 to 0.1) | 0.0 (-0.1 to 0.2) | -0.0 (-0.3 to 0.2) |
| ≥56 | 0.0 (Ref) | 0.1 (-0.1 to 0.3) | 0.1 (-0.1 to 0.3) | 0.1 (-0.1 to 0.3) |

Values reflect coefficients of linear regression analyses in each age stratum, adjusted for OC use, smoking status and BMI. Premenopausal women are the reference category. Green cells indicate p-value <0.001, blue cells indicate p-value <0.05.

Table S6. Differences (95% CI) in adjusted **Glucose** levels (mmol/L) per age stratum, in reference to premenopausal participants.

| **Age (years)** | **Premenopausal (reference)** | **Perimenopausal** | **Naturally postmenopausal** | **Surgically menopausal** |
| --- | --- | --- | --- | --- |
| ≤34 | 0.0 (Ref) | -0.0 (-0.0 to 0.0) | 0.1 (-0.0 to 0.1) | 0.6 (0.3 to 0.9) |
| 35 | 0.0 (Ref) | 0.1 (-0.0 to 0.2) | 0.0 (-0.1 to 0.2) | -0.4 (-1.6 to 0.7) |
| 36 | 0.0 (Ref) | -0.1 (-0.2 to 0.1) | 0.1 (-0.1 to 0.2) | -0.0 (-0.7 to 0.6) |
| 37 | 0.0 (Ref) | -0.0 (-0.1 to 0.1) | 0.1 (-0.1 to 0.2) | -0.2 (-0.5 to 0.1) |
| 38 | 0.0 (Ref) | -0.0 (-0.1 to 0.0) | -0.0 (-0.2 to 0.1) | 0.0 (-0.4 to 0.4) |
| 39 | 0.0 (Ref) | -0.1 (-0.2 to 0.0) | 0.0 (-0.1 to 0.2) | 0.0 (-0.3 to 0.3) |
| 40 | 0.0 (Ref) | 0.0 (-0.1 to 0.1) | -0.0 (-0.2 to 0.1) | -0.3 (-0.8 to 1.3) |
| 41 | 0.0 (Ref) | -0.0 (-0.1 to 0.1) | 0.0 (-0.1 to 0.2) | -0.1 (-0.5 to 0.4) |
| 42 | 0.0 (Ref) | -0.0 (-0.1 to 0.0) | 0.1 (-0.0 to 0.2) | -0.2 (-0.4 to 0.1) |
| 43 | 0.0 (Ref) | 0.1 (-0.0 to 0.1) | 0.1 (-0.0 to 0.2) | -0.1 (-0.3 to 0.2) |
| 44 | 0.0 (Ref) | 0.0 (-0.1 to 0.1) | 0.1 (-0.0 to 0.2) | -0.1 (-0.5 to 0.2) |
| 45 | 0.0 (Ref) | -0.1 (-0.1 to 0.0) | 0.0 (-0.1 to 0.1) | -0.1 (-0.4 to 0.2) |
| 46 | 0.0 (Ref) | -0.0 (-0.1 to 0.0) | 0.1 (-0.0 to 0.1) | -0.1 (-0.3 to 0.1) |
| 47 | 0.0 (Ref) | 0.0 (-0.0 to 0.1) | -0.0 (-0.1 to 0.1) | 0.3 (0.1 to 0.6) |
| 48 | 0.0 (Ref) | -0.0 (-0.1 to 0.0) | -0.1 (-0.2 to -0.0) | -0.0 (-0.2 to 0.2) |
| 49 | 0.0 (Ref) | -0.0 (-0.1 to 0.1) | -0.0 (-0.1 to 0.1) | -0.1 (-0.3 to 0.1) |
| 50 | 0.0 (Ref) | -0.0 (-0.1 to 0.0) | -0.0 (-0.1 to 0.1) | -0.1 (-0.3 to 0.1) |
| 51 | 0.0 (Ref) | -0.1 (-0.2 to 0.0) | -0.1 (-0.1 to 0.0) | 0.1 (-0.1 to 0.4) |
| 52 | 0.0 (Ref) | -0.0 (-0.2 to 0.1) | -0.0 (-0.2 to 0.1) | -0.0 (-0.3 to 0.3) |
| 53 | 0.0 (Ref) | -0.1 (-0.3 to 0.2) | 0.1 (-0.1 to 0.2) | -0.1 (-0.5 to 0.2) |
| 54 | 0.0 (Ref) | 0.0 (-0.2 to 0.2) | -0.0 (-0.2 to 0.2) | -0.1 (-0.4 to 0.1) |
| 55 | 0.0 (Ref) | 0.1 (-0.3 to 0.4) | -0.0 (-0.3 to 0.3) | -0.1 (-0.5 to 0.3) |
| ≥56 | 0.0 (Ref) | 0.1 (-0.3 to 0.5) | 0.0 (-0.3 to 0.4) | 0.1 (-0.3 to 0.5) |

Values reflect coefficients of linear regression analyses in each age stratum, adjusted for OC use, smoking status and BMI. Premenopausal women are the reference category. Green cells indicate p-value <0.001, blue cells indicate p-value <0.05.

Table S7. **Proportional** differences (95% CI) in adjusted **_log_Triglyceride** levels (mmol/L) per age stratum, in reference to premenopausal participants.

| **Age (years)** | **Premenopausal (reference)** | **Perimenopausal** | **Naturally postmenopausal** | **Surgically menopausal** |
| --- | --- | --- | --- | --- |
| ≤34 | 0.0 (Ref) | 0.9 (0.9 to 0.9) | 0.8 (0.7 to 0.8) | 1.0 (0.8 to 1.3) |
| 35 | 0.0 (Ref) | 0.9 (0.8 to 1.0) | 0.8 (0.7 to 0.9) | 1.5 ( 0.7 to 3.4) |
| 36 | 0.0 (Ref) | 0.9 (0.9 to 1.0) | 0.8 (0.8 to 0.9) | 1.0 (0.7 to 1.4) |
| 37 | 0.0 (Ref) | 1.0 (0.9 to 1.1) | 0.8 (0.7 to 0.8) | 1.4 (1.0 to 1.9) |
| 38 | 0.0 (Ref) | 0.9 (0.9 to 1.0) | 0.7 (0.7 to 0.8) | 0.9 (0.7 to 1.3) |
| 39 | 0.0 (Ref) | 1.0 (0.9 to 1.0) | 0.9 (0.8 to 0.9) | 1.0 (0.8 to 1.3) |
| 40 | 0.0 (Ref) | 1.0 (0.9 to 1.0) | 0.8 (0.7 to 0.9) | 1.3 (0.9 to 1.7) |
| 41 | 0.0 (Ref) | 1.0 (0.9 to 1.0) | 0.9 (0.8 to 1.0) | 1.1 (0.9 to 1.4) |
| 42 | 0.0 (Ref) | 1.0 (0.9 to 1.0) | 0.9 (0.8 to 1.0) | 1.1 (0.9 to 1.4) |
| 43 | 0.0 (Ref) | 1.0 (0.9 to 1.0) | 0.9 (0.8 to 1.0) | 1.1 (0.9 to 1.3) |
| 44 | 0.0 (Ref) | 1.0 (0.9 to 1.0) | 0.9 (0.8 to 1.0) | 1.1 (0.9 to 1.3) |
| 45 | 0.0 (Ref) | 1.0 (0.9 to 1.0) | 0.9 (0.9 to 1.0) | 1.1 (0.9 to 1.3) |
| 46 | 0.0 (Ref) | 1.0 (0.9 to 1.0) | 0.9 (0.9 to 1.0) | 1.1 (0.9 to 1.2) |
| 47 | 0.0 (Ref) | 1.0 (1.0 to 1.0) | 1.0 (0.9 to 1.0) | 1.1 (0.9 to 1.2) |
| 48 | 0.0 (Ref) | 1.0 (0.9 to 1.0) | 1.0 (1.0 to 1.0) | 1.1 (0.9 to 1.2) |
| 49 | 0.0 (Ref) | 0.9 (0.9 to 1.0) | 1.0 (0.9 to 1.0) | 1.1 (1.0 to 1.2) |
| 50 | 0.0 (Ref) | 1.0 (0.9 to 1.0) | 1.0 (1.0 to 1.1) | 1.1 (1.0 to 1.3) |
| 51 | 0.0 (Ref) | 0.9 (0.9 to 1.0) | 1.0 (0.9 to 1.0) | 1.1 (0.9 to 1.2) |
| 52 | 0.0 (Ref) | 1.1 (1.0 to 1.2) | 1.0 (0.9 to 1.1) | 1.2 (1.0 to 1.5) |
| 53 | 0.0 (Ref) | 0.8 (0.7 to 1.0) | 0.9 (0.8 to 1.0) | 0.9 (0.7 to 1.1) |
| 54 | 0.0 (Ref) | 1.0 (0.8 to 1.1) | 1.0 (0.8 to 1.1) | 1.0 (0.8 to 1.2) |
| 55 | 0.0 (Ref) | 1.0 (0.8 to 0.2) | 0.9 (0.8 to 1.1) | 1.0 (0.8 to 1.3) |
| ≥56 | 0.0 (Ref) | 0.98 (0.6 to 1.0) | 0.8 (0.7 to 1.0) | 0.8 (0.7 to 1.0) |

Values reflect coefficients of linear regression analyses in each age stratum, adjusted for OC use, smoking status and BMI. Premenopausal women are the reference category. Red cells indicate p-value <0.0001, green cells indicate p-value <0.001, blue cells indicate p-value <0.05.

Table S8. Differences (95% CI) in adjusted **BMI** levels (kg/m^2^) per age stratum, in reference to premenopausal participants.

| **Age (years)** | **Premenopausal (reference)** | **Perimenopausal** | **Naturally postmenopausal** | **Surgically menopausal** |
| --- | --- | --- | --- | --- |
| ≤34 | 0.0 (Ref) | 0.5 (0.3 to 0.8) | 1.1 (0.6 to 1.6) | 2.9 (0.3 to 5.5) |
| 35 | 0.0 (Ref) | -0.1 (-1.0 to 0.8) | 0.5 (-0.8 to 1.8) | 1.9 (-7.1 to 10.9) |
| 36 | 0.0 (Ref) | 0.8 (-0.0 to 1.6) | 0.3 (-0.8 to 1.5) | 1.1 (-3.0 to 5.2) |
| 37 | 0.0 (Ref) | 0.4 (-0.4 to 1.3) | 0.9 (-0.2 to 2.0) | 3.7 (0.4 to 7.0) |
| 38 | 0.0 (Ref) | 0.2 (-0.6 to 1.0) | 1.2 (-0.1 to 2.4) | 0.7 (-3.2 to 4.5) |
| 39 | 0.0 (Ref) | 0.6 (-0.2 to 1.4) | -0.2 (-1.2 to 0.7) | 3.7 (1.3 to 6.2) |
| 40 | 0.0 (Ref) | 0.5 (-0.2 to 1.2) | 0.7 (-0.3 to 1.7) | 7.8 (4.3 to 11.3) |
| 41 | 0.0 (Ref) | 0.4 (-0.2 to 1.1) | 0.2 (-0.7 to 1.2) | 0.4 (-2.4 to 3.2) |
| 42 | 0.0 (Ref) | 0.6 (0.0 to 1.3) | -0.1 (-1.0 to 0.7) | 0.7 (-1.6 to 2.9) |
| 43 | 0.0 (Ref) | 0.5 (-0.0 to 1.0) | 0.2 (-0.6 to 1.1) | 1.2 (-0.8 to 3.2) |
| 44 | 0.0 (Ref) | 0.2 (-0.3 to 0.7) | 0.4 (-0.4 to 1.2) | 2.4 (0.3 to 4.5) |
| 45 | 0.0 (Ref) | 0.0 (-0.4 to 0.5) | 0.5 (-0.3 to 1.3) | 2.1 (0.3 to 0.9) |
| 46 | 0.0 (Ref) | 0.9 (0.4 to 1.3) | 0.7 (0.1 to 1.4) | 0.8 (-0.6 to 2.2) |
| 47 | 0.0 (Ref) | 0.5 (0.0 to 0.9) | 0.2 (-0.4 to 0.8) | 1.2 (-0.3 to 2.6) |
| 48 | 0.0 (Ref) | 0.0 (-0.4 to 0.4) | 0.1 (-0.5 to 0.6) | 1.2 (-0.2 to 2.6) |
| 49 | 0.0 (Ref) | 0.6 (0.1 to 1.0) | 0.2 (-0.3 to 0.7) | 1.0 (-0.1 to 2.2) |
| 50 | 0.0 (Ref) | 0.4 (-0.1 to 0.8) | 0.0 (-0.4 to 0.5) | 1.0 (-0.2 to 2.2) |
| 51 | 0.0 (Ref) | 0.1 (-0.5 to 0.6) | -0.0 (-0.6 to 0.5) | 0.2 (-1.3 to 1.7) |
| 52 | 0.0 (Ref) | 0.7 (-0.3 to 1.6) | 0.3 (-0.6 to 1.2) | 2.2 (0.1 to 4.4) |
| 53 | 0.0 (Ref) | 0.3 (-1.0 to 1.6) | -0.1 (-1.3 to 1.1) | -0.1 (-2.2 to 2.0) |
| 54 | 0.0 (Ref) | -0.2 (-1.6 to 1.2) | -0.2 (-1.5 to 1.1) | 0.4 (-1.5 to 2.3) |
| 55 | 0.0 (Ref) | 0.6 (-1.4 to 2.5) | 0.3 (-1.3 to 2.0) | 1.1 (-1.3 to 3.4) |
| ≥56 | 0.0 (Ref) | -1.4 (-3.6 to 0.7) | -1.2 (-3.1 to 0.6) | -0.5 (-2.4 to 1.5) |

Values reflect coefficients of linear regression analyses in each age stratum, adjusted for OC use and smoking status. Premenopausal women are the reference category. Red cells indicate p-value <0.0001, green cells indicate p-value <0.001.
