# Supplementary figures and images for: Unraveling the associations of age and menopause with cardiovascular risk factors in a large population-based study
Source: BMC Med. 2017 Jan 4;15:2. doi: 10.1186/s12916-016-0762-8 (PMC5210309; doi:10.1186/s12916-016-0762-8)

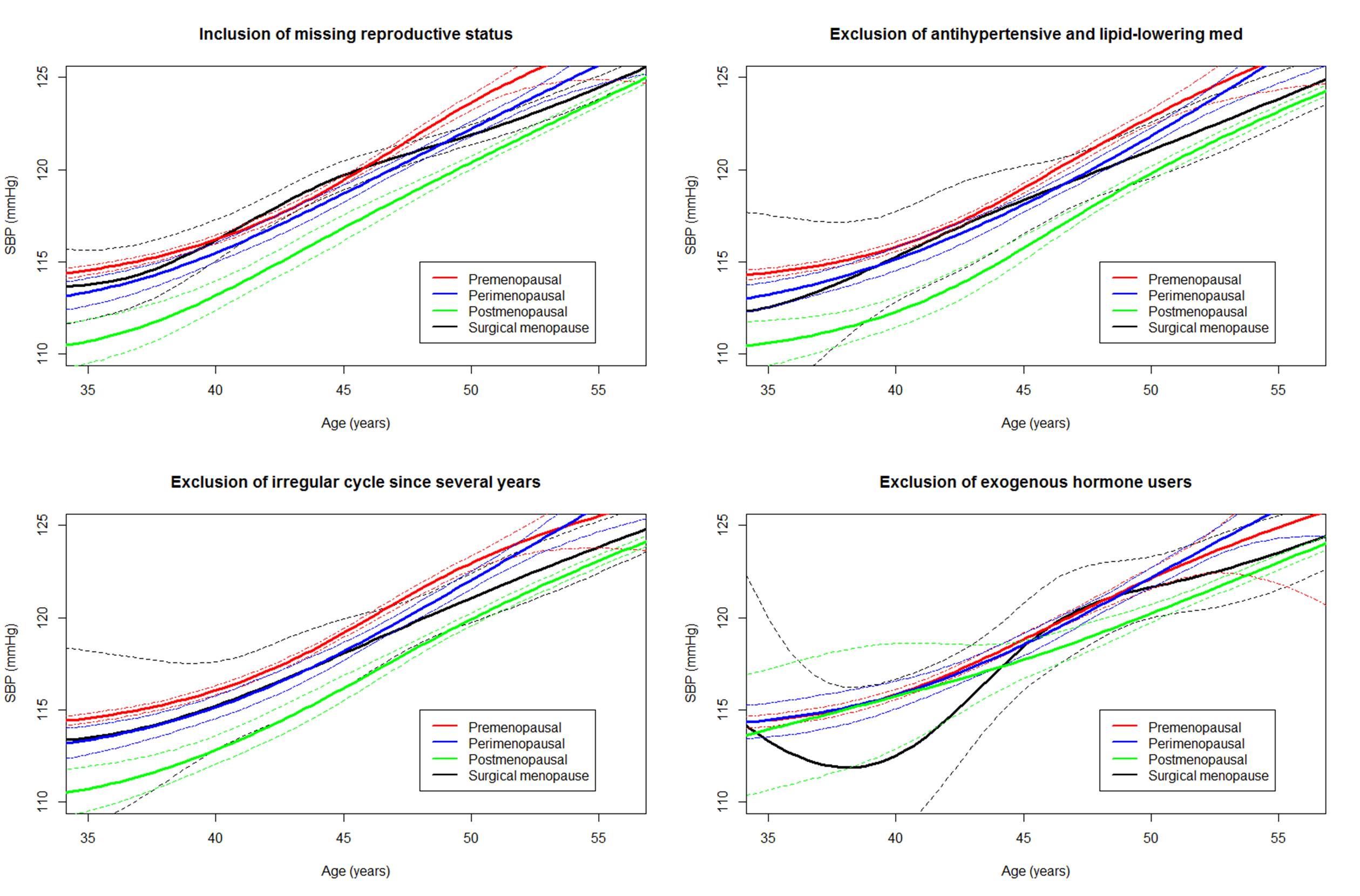

Supplement: Additional file 2: — Figure S1. Sensitivity analyses of associations of systolic blood pressure with age per menopausal status group. From left to right: analyses with inclusion of women with missing reproductive status; analyses with exclusion of women using antihypertensive or lipid-lowering drugs; analyses with exclusion of women with an irregular cycle since several months or years; analyses with exclusion of women using exogenous hormones. (JPG 337 kb) [file 12916_2016_762_MOESM2_ESM.jpg]

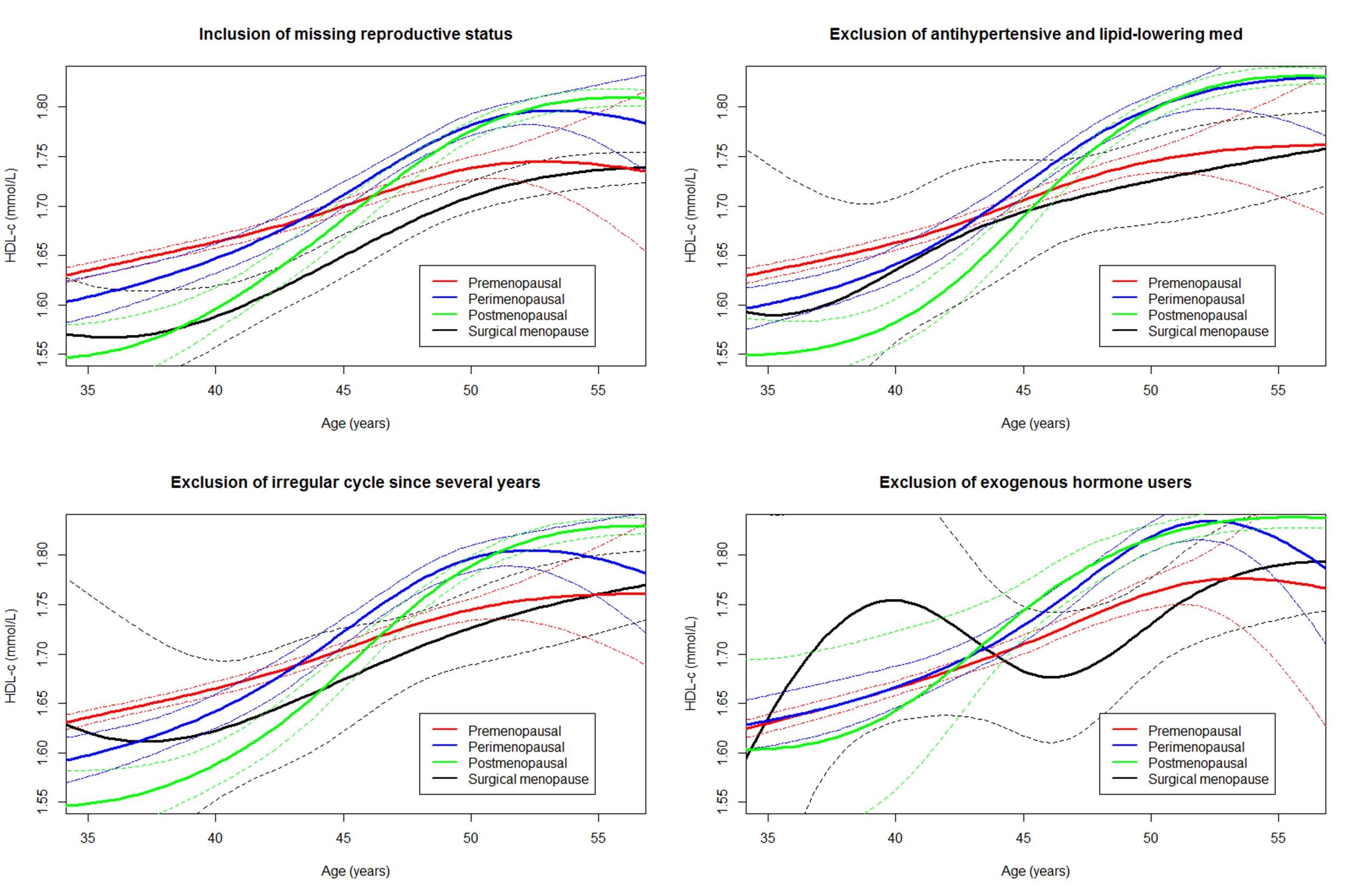

Supplement: Additional file 3: — Figure S2. Sensitivity analyses of associations of diastolic blood pressure with age per menopausal status group. From left to right: analyses with inclusion of women with missing reproductive status; analyses with exclusion of women using antihypertensive or lipid-lowering drugs; analyses with exclusion of women with an irregular cycle since several months or years; analyses with exclusion of women using exogenoushormones. (JPG 640 kb) [file 12916_2016_762_MOESM3_ESM.jpg]

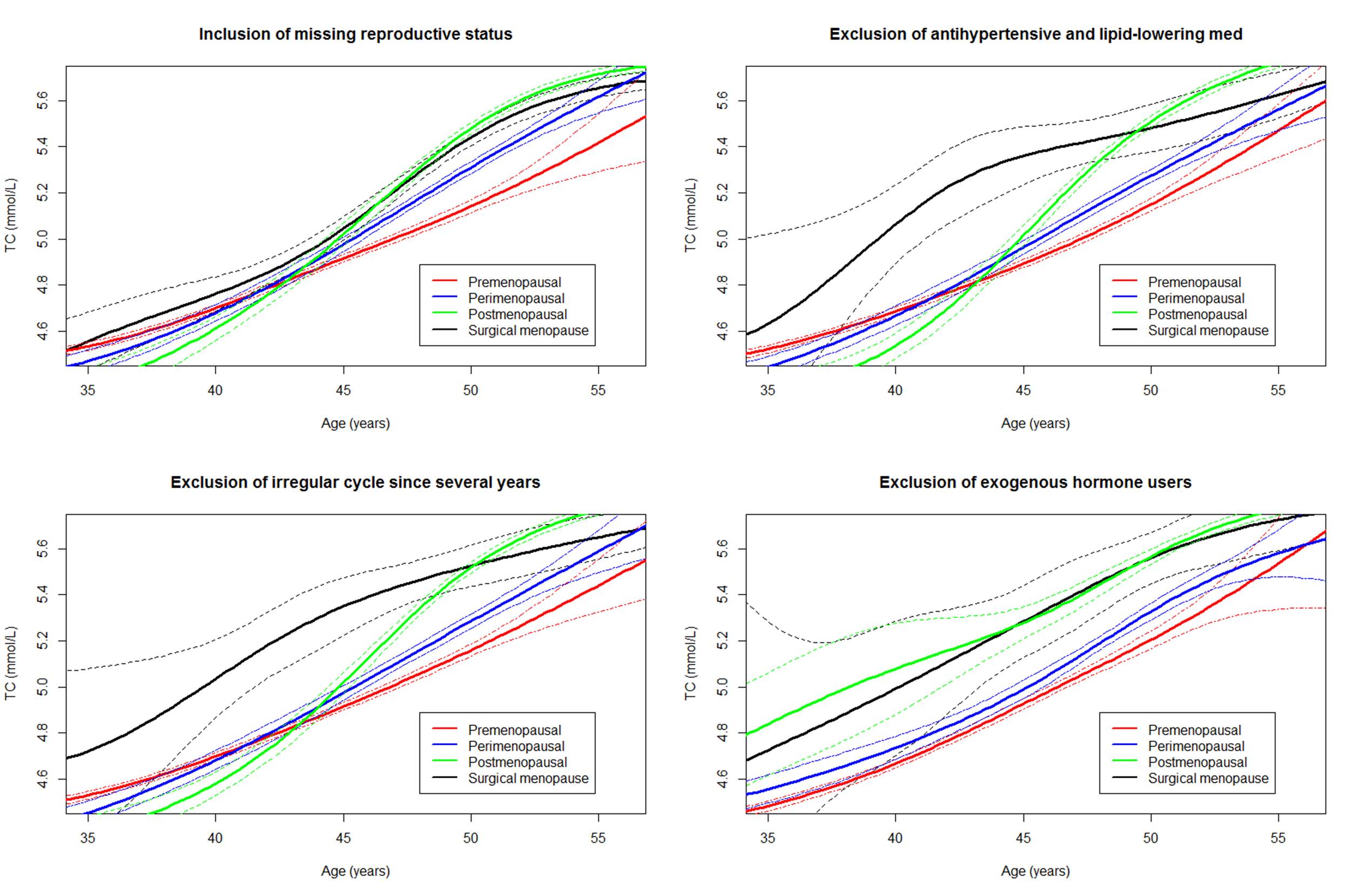

Supplement: Additional file 4: — Figure S3. Sensitivity analyses of associations of total cholesterol with age per menopausal status group. From left to right: analyses with inclusion of women with missing reproductive status; analyses with exclusion of women using antihypertensive or lipid-lowering drugs; analyses with exclusion of women with an irregular cycle since several months or years; analyses with exclusion of women using exogenous hormones. (JPG 350 kb) [file 12916_2016_762_MOESM4_ESM.jpg]

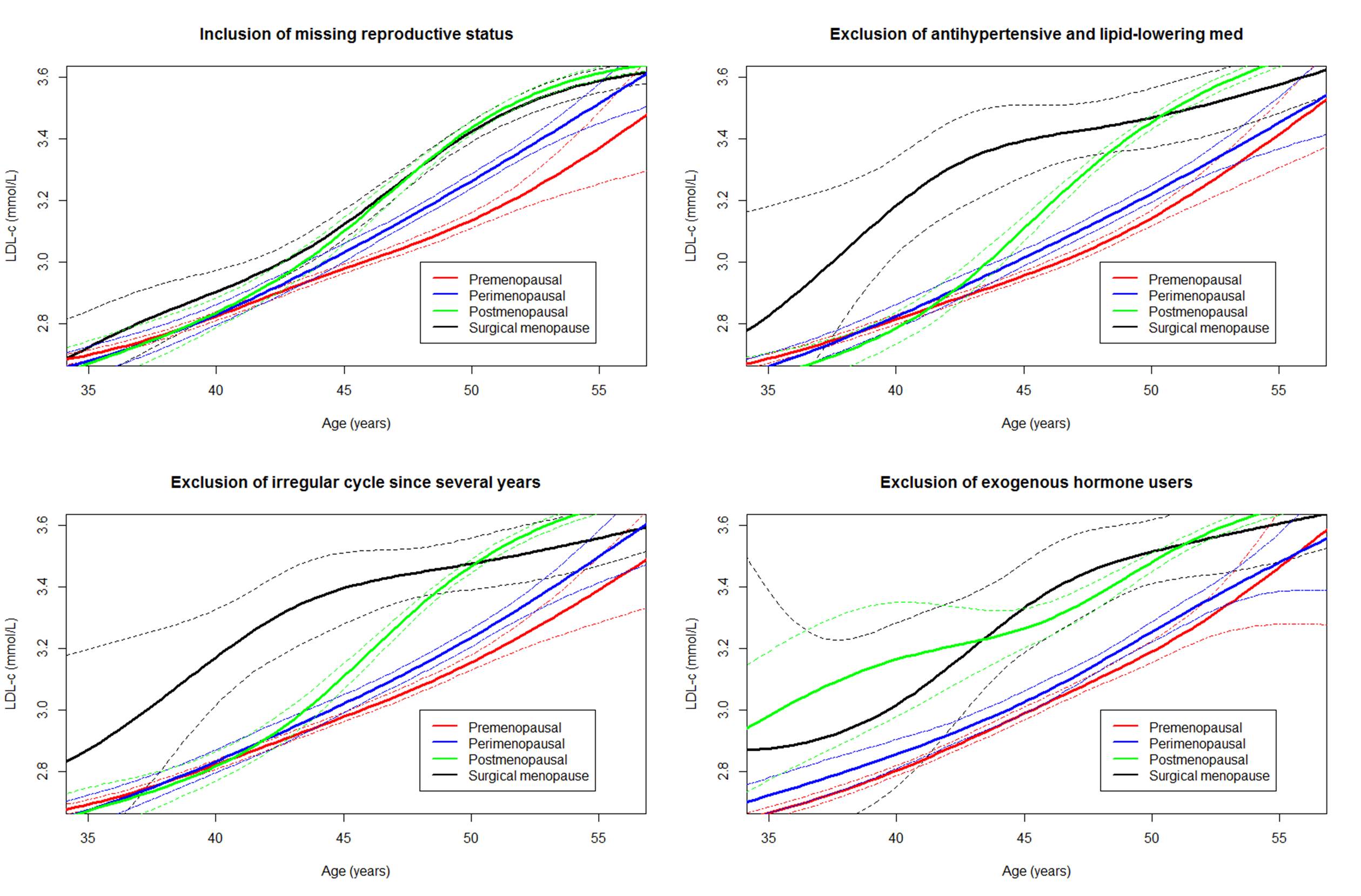

Supplement: Additional file 5: — Figure S4. Sensitivity analyses of associations of low-density lipoprotein cholesterol with age per menopausal status group. From left to right: analyses with inclusion of women with missing reproductive status; analyses with exclusion of women using antihypertensive or lipid-lowering drugs; analyses with exclusion of women with an irregular cycle since several months or years; analyses with exclusion of women using exogenous hormones. (JPG 352 kb) [file 12916_2016_762_MOESM5_ESM.jpg]

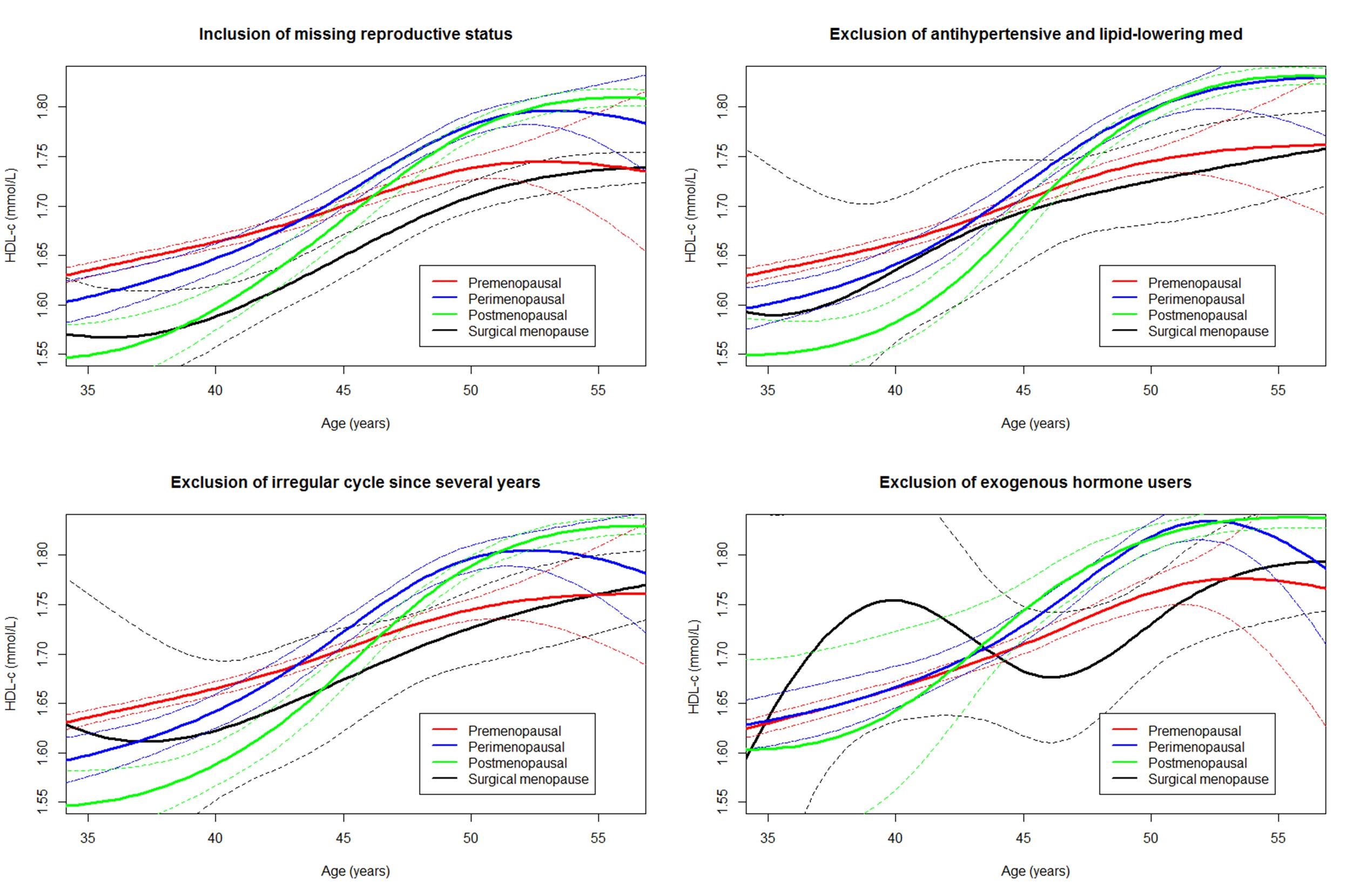

Supplement: Additional file 6: — Figure S5. Sensitivity analyses of associations of high-density lipoprotein cholesterol with age per menopausal status group. From left to right: analyses with inclusion of women with missing reproductive status; analyses with exclusion of women using antihypertensive or lipid-lowering drugs; analyses with exclusion of women with an irregular cycle since several months or years; analyses with exclusion of women using exogenous hormones. (JPG 640 kb) [file 12916_2016_762_MOESM6_ESM.jpg]

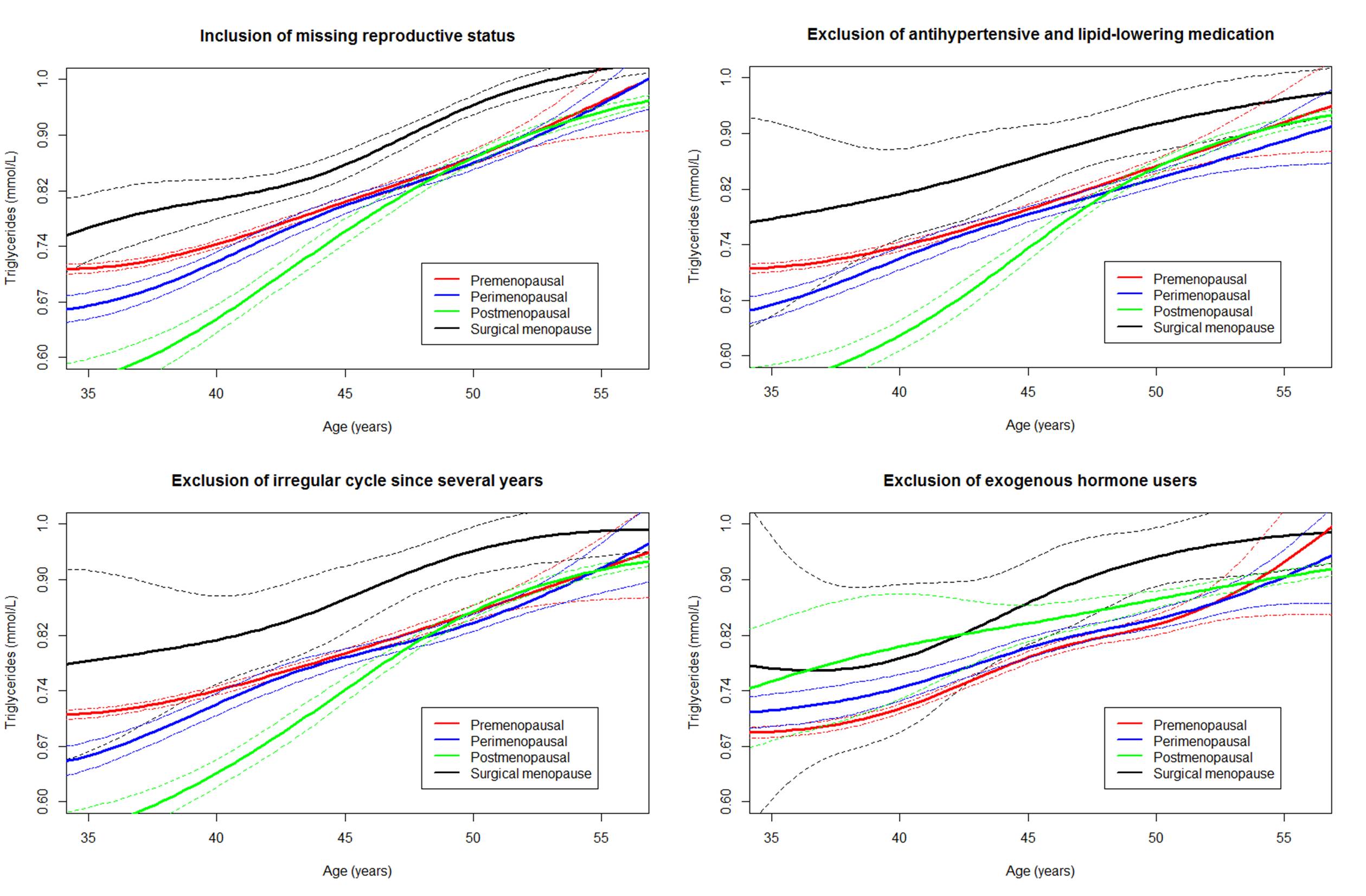

Supplement: Additional file 7: — Figure S6. Sensitivity analyses of associations of glucose with age per menopausal status group. From left to right: analyses with inclusion of women with missing reproductive status; analyses with exclusion of women using antihypertensive or lipid-lowering drugs; analyses with exclusion of women with an irregular cycle since several months or years; analyses with exclusion of women using exogenous hormones. (JPG 345 kb) [file 12916_2016_762_MOESM7_ESM.jpg]

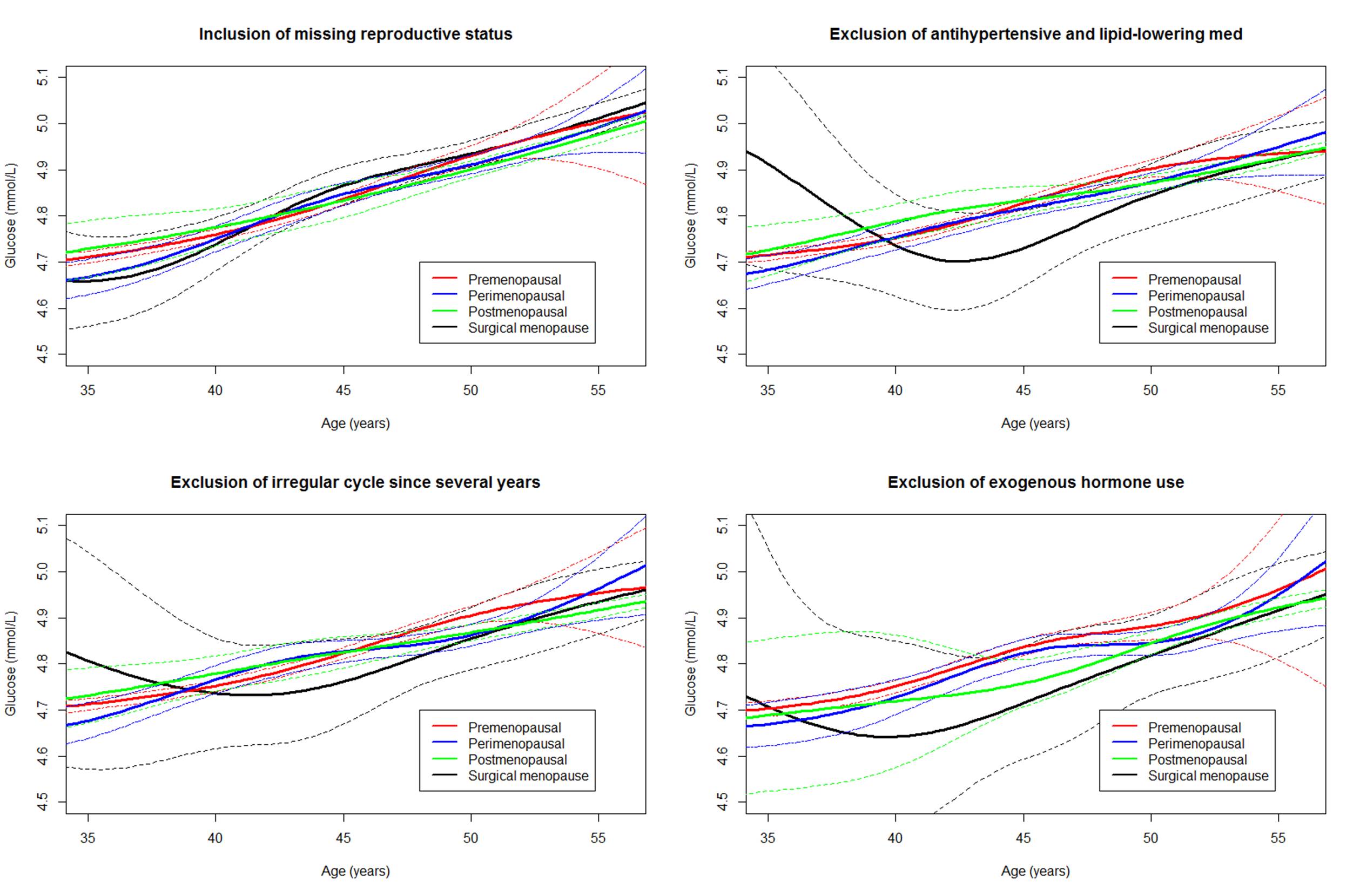

Supplement: Additional file 8: — Figure S7. Sensitivity analyses of associations of triglycerides with age per menopausal status group. From left to right: analyses with inclusion of women with missing reproductive status; analyses with exclusion of women using antihypertensive or lipid-lowering drugs; analyses with exclusion of women with an irregular cycle since several months or years; analyses with exclusion of women using exogenous hormones. (JPG 324 kb) [file 12916_2016_762_MOESM8_ESM.jpg]

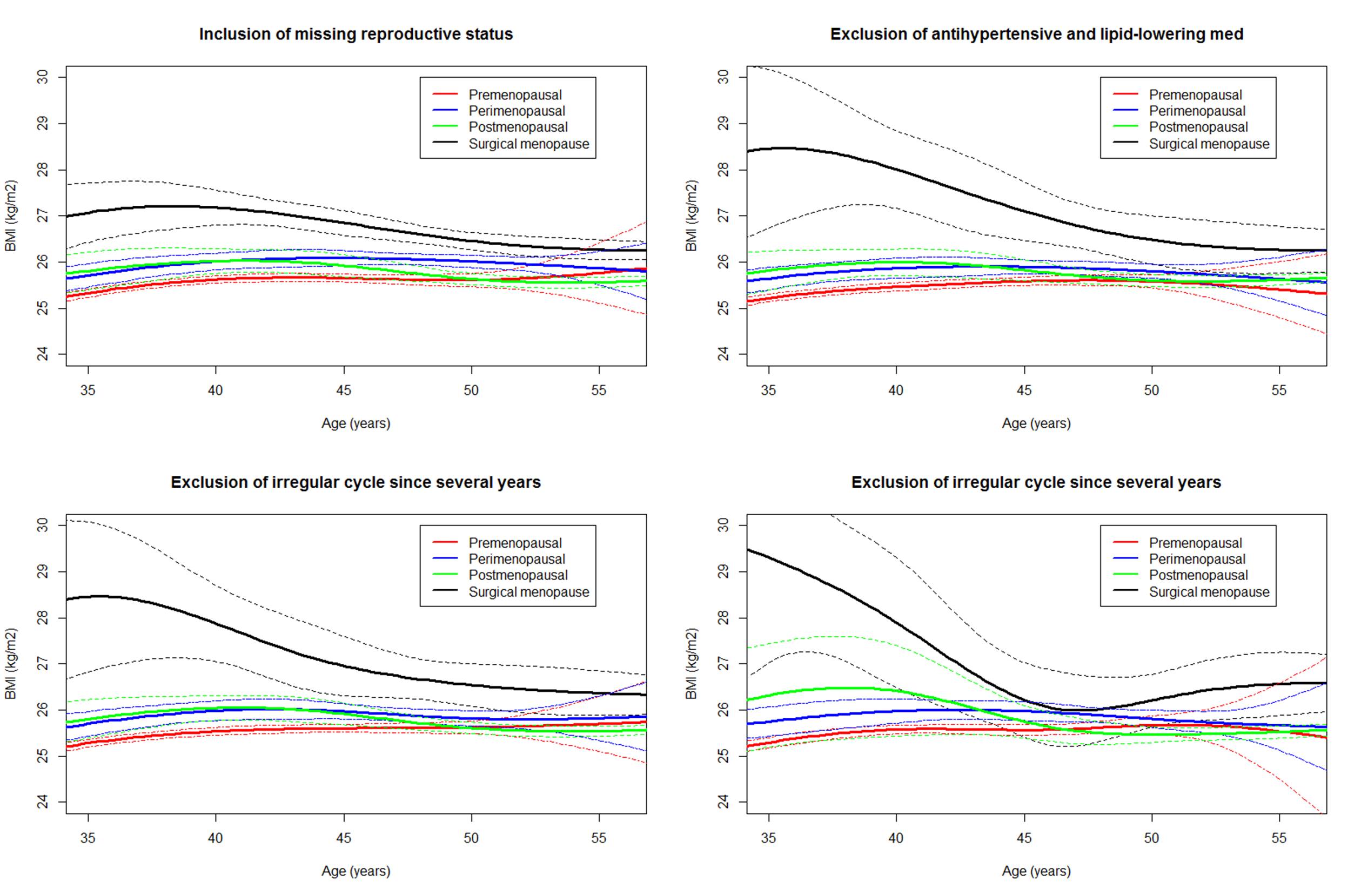

Supplement: Additional file 9: — Figure S8. Sensitivity analyses of associations of body mass index with age per menopausal status group. From left to right: analyses with inclusion of women with missing reproductive status; analyses with exclusion of women using antihypertensive or lipid-lowering drugs; analyses with exclusion of women with an irregular cycle since several months or years; analyses with exclusion of women using exogenous hormones. (JPG 315 kb) [file 12916_2016_762_MOESM9_ESM.jpg]
